# Trends in nulliparous singleton alive births by cesarean section in India: Empirical patterns across public and private hospitals for 720 districts, 2016–2021

**DOI:** 10.1371/journal.pgph.0005501

**Published:** 2025-11-25

**Authors:** Sunil Rajpal, Shreya Ronanki, Nehantha Sathesh, Rockli Kim, S. V. Subramanian

**Affiliations:** 1 Department of Economics, FLAME University, Pune, India; 2 Centre for Research in Wellbeing and Happiness, FLAME University, Pune, India; 3 Division of Health Policy and Management, College of Health Science, Korea University, Seoul, South Korea; 4 Harvard Center for Population and Development Studies, Cambridge, Massachusetts, United States of America; 5 Department of Social and Behavioral Sciences, Harvard T.H. Chan School of Public Health, Boston, Massachusetts, United States of America; PLOS: Public Library of Science, UNITED STATES OF AMERICA

## Abstract

Unnecessary cesarean surgeries pose significant short and long-term risks, affecting fertility, future pregnancies, and child health outcomes. Timely monitoring and precise targeting are crucial to mitigate additional health and economic burdens. This study examines trends and patterns in cesarean deliveries among nulliparous singleton births across all 36 states and 720 districts in India, comparing public and private hospitals between 2016 and 2021. Using a repeated cross-sectional analysis of two waves of India’s National Family Health Survey, we assess the prevalence of cesarean births and explore how the relative contribution of different geographical levels (villages/blocks, districts, states) to the total variation has evolved over time. At the national level, cesarean rates in public facilities declined by 1.2 percentage points, while private facilities saw an increase of 2.1 percentage points. Regional disparities were evident, with Telangana and Jammu & Kashmir consistently reporting the highest cesarean rates in both sectors. Variation across states was more pronounced than at other geographical levels. In private hospitals, the state-level variance partitioning coefficient increased from 69.2% in 2016 to 78.2% in 2021, whereas in public hospitals, it rose from 44.7% to 48.6% over the same period. Notably, states such as Tamil Nadu, Telangana, and Kerala had nearly all districts falling into the high-prevalence category. It is important to consider burden and variation among smaller geographical units (like districts) to monitor the burden. Increased inter-state inequalities with high prevalence among private facilities in southern states imply the absence of uniform protocols for cesarean births. This calls for urgent policy action to regulate the healthcare sector about the issue, and more awareness is required to avoid the additional health and economic burden.

## Introduction

Cesarean section procedures of childbirth are proven to be effective in mitigating mortality and morbidity risks in the presence of maternal or fetal complications [1]. With overtime research and practice, cesarean births are now considered as safe as vaginal delivery and are also opted for, even in the absence of any medical or obstetric indication [2,3]. The global average for cesarean deliveries increased from 12.1% in 2000 to 21.2% in 2015 [1]. While cesarean delivery rates were initially observed to be more prevalent in high-income countries [4], last two decades have shown a soaring trend for developing and populous countries. For example, the national average for cesarean birth rates in India has doubled from 7.1% to 17.2% between 2006 and 2016 [5,6]. Whereas, WHO guidelines in 1985 recommended maintaining national average rates between 10% and 15% [7]. Given the short- and long-term effects of unnecessary cesarean surgeries (on fertility, future pregnancies, and child health outcomes) [8], timely monitoring and precise targeting are crucial to avoid additional health and economic burdens.

An important feature of cesarean childbirth in India is the huge public-private divide. While about half (48%) of childbirths in private facilities are conducted through cesarean procedures, only 14% of public deliveries are surgical in India [9]. Such skewness suggests a potential moral hazard, i.e., hospitals responding to financial incentives in their treatment choices [10]. Previous studies have also observed a strong association between cesarean deliveries and private healthcare providers [11,12]. Consequently, patients are forced to bear a substantial out-of-pocket expenditure in the absence of regulatory guidelines for service fees charged by private hospitals. Further, financial cover for cesarean births in private facilities does not exist in government schemes except *Janani Suraksha Yojana* or *Ayushman Bharat* [13]. Hence, nonessential surgeries can also be burdensome from an economic standpoint with non-trivial equity implications, and it is crucial to direct policy focus toward the subject matter.

Since cesarean section deliveries are not supposed to be very high or very low, but should be within a range, it becomes important to consider inequalities across population sub-groups. Previous papers have observed substantial inter-state variations, for example, as per the NFHS 2021, the proportion of cesarean to total institutional births was recorded at 81.5% for private hospitals in Telangana, but only 21% in Nagaland. However, such aggregate statistics may not inform about the prevalence and variation within states, across smaller units like districts and villages. To maintain a national average within range, it is critical to monitor the progress in improvements at smaller geographical levels.

This paper compares the trends and patterns in nulliparous singleton cesarean births across 720 districts in India for public and private hospitals separately between 2016 and 2021. The primary objective of the paper is to examine the district-level trends and patterns in the cesarean section births for all 720 districts in India for public and private hospitals separately. In addition, the study also attempts to quantify the overtime change in variation in c-section births (public and private facilities) across administrative geographies in India. The analysis can offer valuable insights for precisely targeting and prioritizing the high burden areas. We restricted our sample to nulliparous mothers as first-order births are considered the baseline for the future c-section risk, and therefore, preventing the first c-section birth is the key to reducing overall burden. Further, from an obstetric history standpoint, nulliparous mothers with a single cephalic pregnancy are better comparable to those having a repeated (successive) c-section birth. Employing multilevel regression models, we compared the variation across the three geographies (states, districts, and clusters) and how it has changed between the two time periods. While previous studies have examined the district-level trends and patterns in cesarean birth rates, none of them have analysed how the small area units have progressed over time.

## Methods

### Ethics statement

The NFHS obtained data with informed consent, and the survey protocol, including all questionnaire content, received approval from the Institutional Review Boards of the International Institute for Population Studies and ICF. For this study, the Harvard Longwood Campus Institutional Review Board (IRB) permits researchers to self-assess whether their work requires IRB oversight using the IRB Decision Tool. Based on this tool, our analysis did not qualify as human participant research under regulatory definitions, and thus was deemed exempt from full institutional review.

### Overview

We utilized data from the fourth and fifth rounds of the National Family Health Survey (equivalent to the Demographic Health Survey) conducted in 2015–16. Initiated in the early 1990s, the NFHS is part of the Demographic Health Surveys (DHS) program and provides individual-level information on important population, health, and nutrition indicators. Since its inception, DHS has conducted more than 400 surveys in 90 countries.

### Data

The NFHS adopts a multistage, stratified cluster sampling design. Microdata from the fourth and fifth rounds of the surveys are the latest available and were used in this study. The NFHS collects the data for rural and urban areas separately using the latest census data as the sampling frame. According to the Demographic Health Survey, the clusters, which are villages (for rural areas) and Census Enumeration Blocks (CEBs) (for urban areas), serve as primary sampling units (PSU). A representative sample of households was constructed for rural areas via stratified, probabilistic two-stage random sampling. In the first stage, the PSU (or cluster) corresponding to villages was classified on key variables and selected by probability proportional to cluster size. This was followed by household selections from the household list using systematic sampling with equal probability [5,14]. A similar process was used for the urban areas, but because the urban clusters correspond to census enumeration blocks, a mix of a two-stage sampling approach was employed. It may be noted that PSUs with more than 300 households are divided into 100–150 household segments. Hence one cluster can be a PSU or a segment of PSU.

### Primary outcomes and geographies

The primary outcome variable was binary (Yes = 1/No = 0) of cesarean section births across all public and private facilities. The analyses include estimates for 720 districts nested within 36 states and union territories (see Fig A in S1 Text for sampl\e construction details)

### Statistical analyses

We employed a four-level logistic regression model to partition the total variation in the nulliparous singleton cesarean births across public and private hospitals (level-1); cluster *j* (level-2); district *k* (level-3); state *l* (level-4): $Y_{ijkl}=\;\beta_{\text{0}}+(u_{\text{0}jkl}+\;v_{\text{0}kl}+\;f_{\text{0}l})$. In the model mentioned above, $u_{\text{0}jkl}$, $v_{\text{0}kl}$, $f_{\text{0}l}$ are model residuals specific to cluster, district, and state, respectively. These set of residuals are assumed to have a normal distribution around the mean of 0 and the variance of $u_{\text{0}jkl}$ ~ (0, $\sigma_{\text{u0}}^{\text{2}}$); $v_{\text{0}kl}$ ~ (0, $\sigma_{\text{v0}}^{\text{2}}$); $f_{\text{0}l}$ ~ (0, $\sigma_{\text{f0}}^{\text{2}}$). Here, the term $\sigma_{\text{u0}}^{\text{2}}$ denotes within-district, inter-cluster variation, $\sigma_{\text{v0}}^{\text{2}}$ denotes within-state, inter-district variation and $\sigma_{\text{f0}}^{\text{2}}$ stands for inter-state variation. Variance across individual hospitals is not computed directly for binary outcomes and is instead assumed to follow a logistic distribution with a fixed variance of $\pi^{\text{2}}$/3 or 3.29 [15]. We then computed the variance partitioning coefficient to assess the significance of each geographical unit (z) in total variability as ($\frac{\sigma_{z}^{\text{2}}}{\sigma_{u\text{0}}^{\text{2}}+\;\sigma_{v\text{0}}^{\text{2}}+\;\sigma_{f\text{0}}^{\text{2}}}$) * 100. Multilevel modeling was performed using the STATA 15 [16] and MLwiN 3.0 software program (using *runmlwin*) [17] and the Monte Carlo Markov Chain (MCMC) method using the Gibbs sampler, keeping the default prior distribution of Iterated Generalized Least Square (IGLS) as the starting value [18].

Based on the multilevel logistic model estimates, we then generated precision-weighted cluster-level predicted probabilities of cesarean deliveries. For more robust estimates, these probabilities were predicted by pooling information (and borrowing strength) from other clusters that share the same district membership. The probability of each Y for each village/block was calculated as exp(($\beta_{\text{0}}+u_{\text{0}jkl}+\;v_{\text{0}kl}+\;f_{\text{0}l})+(\text{1}/\text{exp}(\beta_{\text{0}}+u_{\text{0}jkl}+\;v_{\text{0}kl}+\;f_{\text{0}l}))$. We then computed within-district, inter-cluster variation (small area variation) in cesarean births via standard deviation (SD) from the above precision-weighted estimates. The shapefiles for creating maps for the 640 districts per the NFHS 4 and NFHS 5 surveys were obtained from the International Institute for Population Sciences (IIPS).

## Results

Table 1 outlines the state-wise prevalence of cesarean section deliveries in public and private hospitals across India for 2016 and 2021. At the national level, cesarean rates in public facilities declined by 1.2 percentage points (from 15.5%; N = 8,278 in 2016 to 14.3%; N = 21,512 in 2021), while private facilities increased by 2.1 percentage points (from 45.4%; N = 11,110 in 2016 to 47.5%; N = 23,614 in 2021). Regional disparities were apparent, with Telangana and Jammu and Kashmir consistently reporting the highest cesarean rates in both sectors. Specifically, in public hospitals, Telangana’s prevalence increased from 43.5% (N = 126) in 2016 to 44.5% (N = 1,623) in 2021, while Jammu and Kashmir rose from 39.9% (N = 874) to 42.7% (N = 2,153). Private hospitals in these states also showed high rates, with Telangana climbing from 78.3% (N = 497) to 81.5% (N = 2,753) and Jammu and Kashmir rising from 79.7% (N = 163) to 82.1% (N = 232). In contrast, socioeconomically weaker northern states like Bihar, Jharkhand, and Uttar Pradesh consistently reported the lowest prevalence in public hospitals. Bihar’s rates fell from 4.4% (N = 155) in 2016 to 3.6% (N = 448) in 2021, while Jharkhand decreased slightly from 7.1% (N = 135) to 7.0% (N = 413), and Uttar Pradesh from 7.2% (N = 399) to 6.2% (N = 1,317). In private facilities, Rajasthan consistently recorded the lowest rates, declining from 28.2% (N = 409) to 27.0% (N = 627). The disparity between states widened over time. In the public sector, the gap between the highest and lowest prevalence increased by 1.8 percentage points (from 39.1% in 2016 to 40.9% in 2021). In the private sector, this disparity grew significantly by 6.1 percentage points (from 53.4% in 2016 to 59.5% in 2021). These patterns were consistent when considering the distribution of cesarean deliveries across public and private sector by states (See Table A in S1 for details).

**Table 1:** Prevalence of birth delivered by cesarean section in public and private hospitals across states, India, NFHS 2016-2021.

	Public						Private						
	2016			2021			2016				2021		
	N	Mean (%)	SE	N	Mean (%)	SE	N	Mean (%)	SE	N		Mean (%)	SE
**All India**	(8278/53349)	15.5	0.16	(21512/150299)	14.3	0.09	(11110/24461)	45.4	0.32	(23614/49746)		47.5	0.22
Andhra Pradesh	(128/446)	28.8	2.15	(377/1421)	26.6	1.17	(459/787)	58.3	1.76	(821/1285)		63.9	1.34
Arunachal Pradesh	(120/883)	13.6	1.15	(710/4177)	17.0	0.58	(62/144)	42.9	4.14	(109/230)		47.3	3.30
Assam	(509/2888)	17.6	0.71	(1214/7991)	15.2	0.40	(343/560)	61.2	2.06	(680/961)		70.8	1.47
Bihar	(155/3497)	4.4	0.35	(448/12352)	3.6	0.17	(596/1659)	35.9	1.18	(1520/3834)		39.6	0.79
Chhattisgarh	(144/1980)	7.3	0.58	(543/6097)	8.9	0.36	(290/594)	48.8	2.05	(593/1039)		57.0	1.54
Goa	(20/101)	19.5	3.96	(67/214)	31.5	3.18	(49/84)	58.3	5.41	(77/154)		50.0	4.04
Gujarat	(159/1025)	15.5	1.13	(551/4457)	12.4	0.49	(510/1645)	31.0	1.14	(1373/4499)		30.5	0.69
Haryana	(179/1702)	10.5	0.74	(472/4027)	11.7	0.51	(281/1069)	26.3	1.35	(838/2467)		34.0	0.95
Himachal Pradesh	(163/850)	19.1	1.35	(327/1875)	17.4	0.88	(92/196)	47.1	3.57	(211/411)		51.4	2.47
Jharkhand	(135/1897)	7.1	0.59	(413/5938)	7.0	0.33	(431/1016)	42.4	1.55	(758/1616)		46.9	1.24
Karnataka	(388/2026)	19.2	0.87	(1273/5642)	22.6	0.56	(511/1147)	44.5	1.47	(1279/2435)		52.5	1.01
Kerala	(105/429)	24.4	2.08	(358/961)	37.2	1.56	(278/702)	39.5	1.85	(702/1752)		40.1	1.17
Madhya Pradesh	(496/6235)	8.0	0.34	(1088/13212)	8.2	0.24	(543/1231)	44.1	1.42	(744/1420)		52.4	1.33
Maharashtra	(296/1893)	15.7	0.84	(1065/5830)	18.3	0.51	(625/1681)	37.2	1.18	(1186/3059)		38.8	0.88
Manipur	(272/1145)	23.7	1.26	(400/1619)	24.7	1.07	(261/526)	49.5	2.18	(300/566)		53.0	2.10
Meghalaya	(66/567)	11.6	1.35	(290/3153)	9.2	0.51	(70/193)	36.1	3.47	(191/465)		41.1	2.28
Mizoram	(176/1120)	15.8	1.09	(174/1781)	9.8	0.70	(40/103)	39.1	4.83	(52/172)		30.1	3.51
Nagaland	(97/499)	19.4	1.77	(84/1042)	8.0	0.84	(46/124)	36.9	4.35	(43/184)		23.6	3.14
Odisha	(573/3603)	15.9	0.61	(1036/6779)	15.3	0.44	(317/560)	56.6	2.10	(700/991)		70.6	1.45
Punjab	(253/1268)	19.9	1.12	(934/3129)	29.9	0.82	(396/979)	40.4	1.57	(1218/2194)		55.5	1.06
Rajasthan	(308/3750)	8.2	0.45	(837/11566)	7.2	0.24	(409/1452)	28.2	1.18	(627/2322)		27.0	0.92
Sikkim	(85/425)	20.0	1.94	(159/525)	30.4	2.01	(33/64)	51.9	6.29	(39/71)		55.4	5.94
Tamil Nadu	(669/2380)	28.1	0.92	(1570/4357)	36.0	0.73	(671/1320)	50.9	1.38	(1346/2103)		64.0	1.05
Tripura	(116/521)	22.2	1.82	(362/1597)	22.7	1.05	(66/88)	74.5	4.67	(148/214)		69.3	3.16
Uttar Pradesh	(399/5507)	7.2	0.35	(1317/21355)	6.2	0.16	(1470/3992)	36.8	0.76	(3280/8337)		39.3	0.54
Uttarakhand	(117/1120)	10.4	0.91	(328/2338)	14.0	0.72	(224/551)	40.6	2.09	(332/768)		43.2	1.79
West Bengal	(342/1595)	21.5	1.03	(939/4100)	22.9	0.66	(423/556)	76.1	1.81	(844/1016)		83.1	1.18
Telangana	(126/290)	43.5	2.92	(1623/3647)	44.5	0.82	(497/635)	78.3	1.64	(2753/3377)		81.5	0.67
Ladakh	(42/247)	16.9	2.39	(197/501)	39.3	2.18	(1/2)	32.9	47.00	(2/2)		100.0	0.00
Puducherry	(101/336)	30.0	2.50	(165/474)	34.8	2.19	(79/175)	45.3	3.77	(121/288)		42.0	2.91
Jammu and Kashmir	(874/2191)	39.9	1.05	(2153/5038)	42.7	0.70	(163/205)	79.7	2.82	(232/283)		82.1	2.28
Lakshadweep	(31/83)	37.7	5.35	(50/179)	28.1	3.37	(25/49)	50.2	7.22	(36/96)		37.7	4.97
Delhi	(124/363)	34.0	2.49	(323/1827)	17.7	0.89	(79/202)	38.9	3.44	(366/850)		43.1	1.70
Chandigarh	(12/55)	21.2	5.57	(43/142)	30.3	3.87	(13/19)	66.0	11.17	(12/26)		44.3	9.93
D & N Haveli; D & Diu	(27/141)	19.4	3.34	(93/537)	17.2	1.63	(57/142)	40.1	4.13	(93/224)		41.6	3.30
A & N Island	(59/291)	20.2	2.36	(99/419)	23.6	2.08	(7/9)	73.7	15.57	(28/35)		79.2	6.96

**Fig 1:**
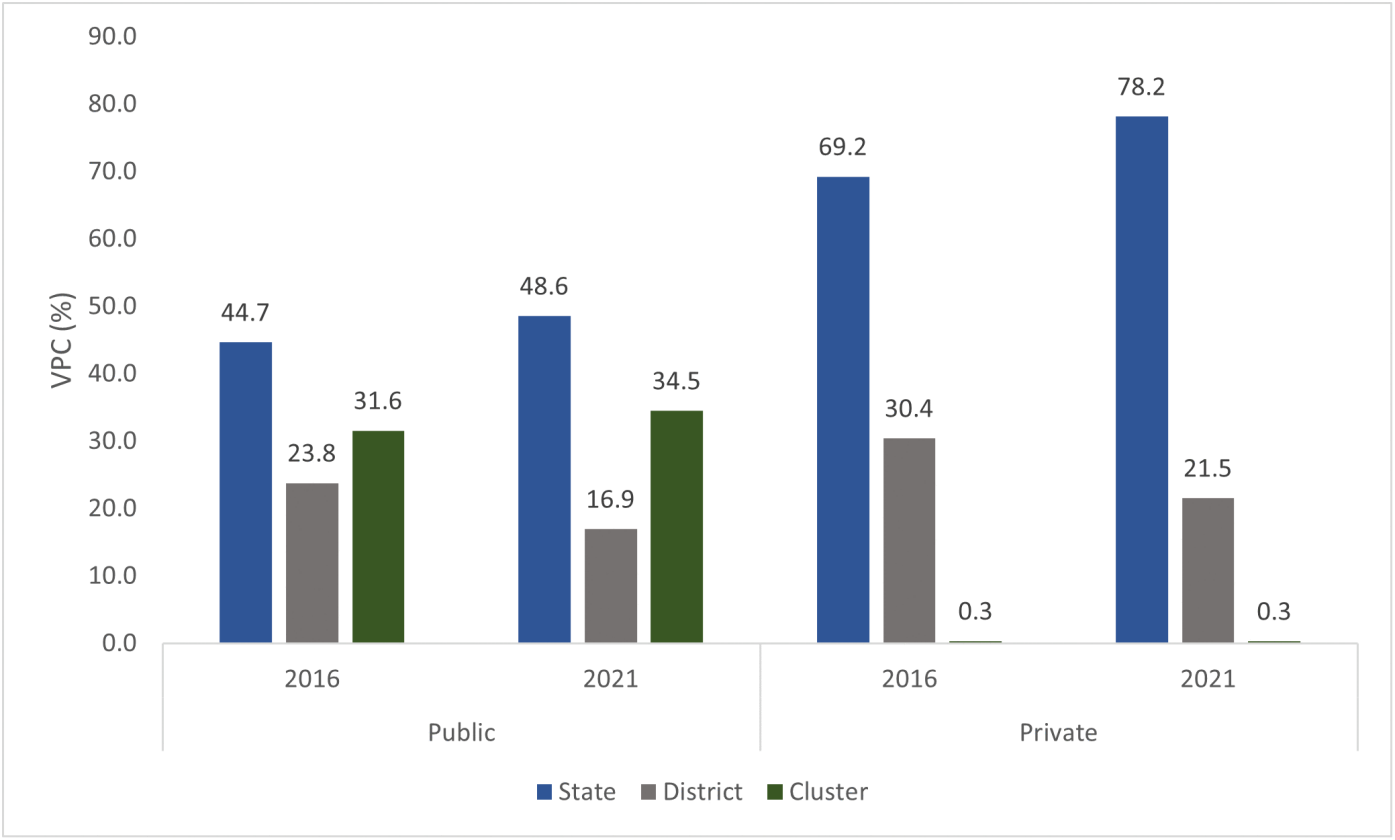
Geographic variance partitioning (%) by clusters, districts, and states for births by cesarean section across public and private facilities, India, NFHS 2016 & 2021.

Results of geographic variance partitioning (Fig 1) revealed that state-level variation in cesarean births was the highest compared to district and cluster-level variations in both public and private sectors. This variation was notably higher in the private sector, where the state-level variance partitioning coefficient increased from 69.2% in 2016 to 78.2% in 2021, compared to the public sector, which rose from 44.7% to 48.6% during the same period.

**Fig 2:**
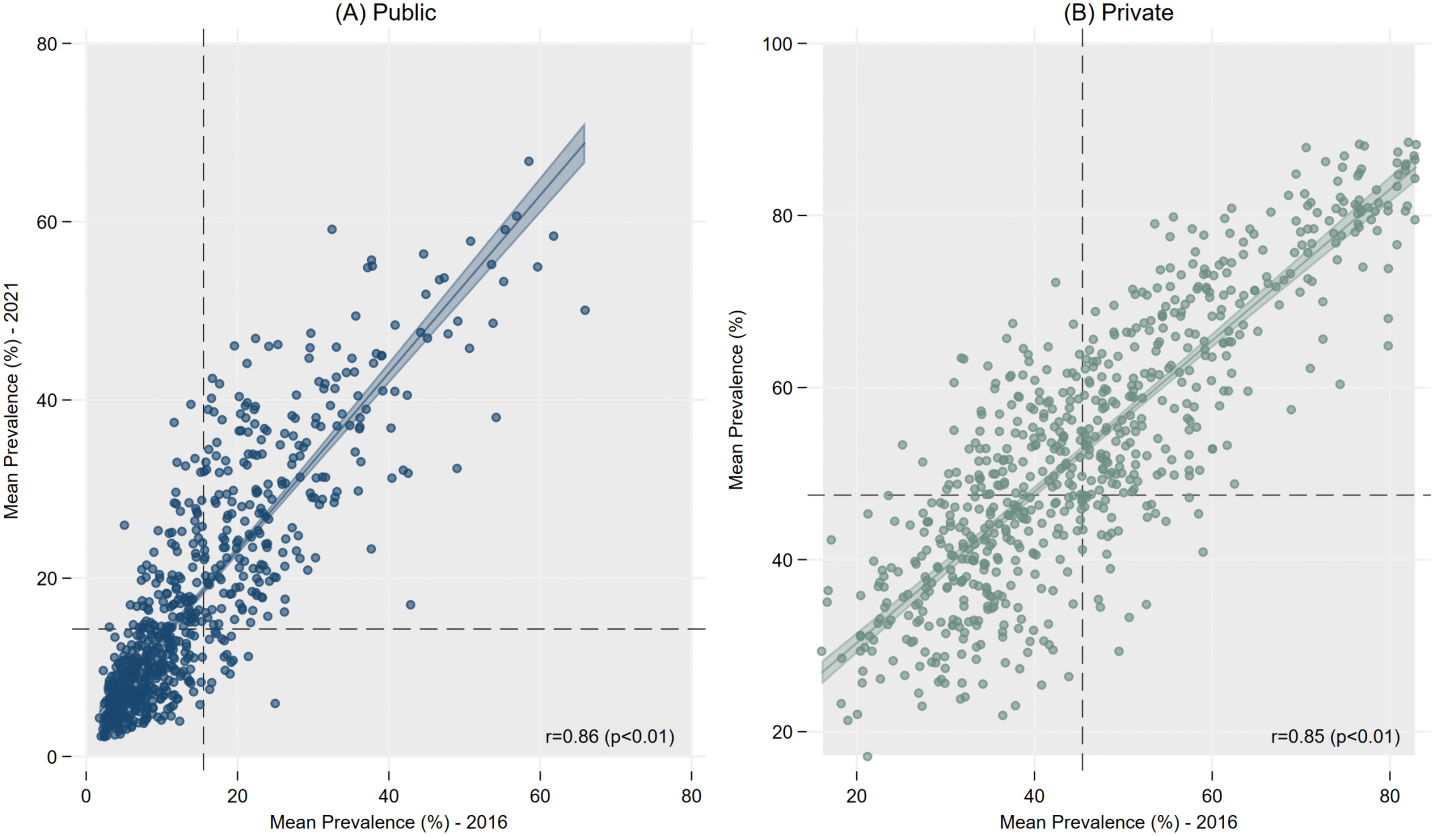
Correlation plots of the district-level mean prevalence (%) for 2016 and 2021 for cesarean section births, India, NFHS 2016 & 2021.

The correlation between the district-level mean prevalence of cesarean births in 2016 and 2021 (Fig 2) was high for both the public (r = 0.86) and private (r = 0.85) sectors, indicating a strong relationship between the rates of cesarean births in 2016 to those in 2021, irrespective of the sector.

**Fig 3:**
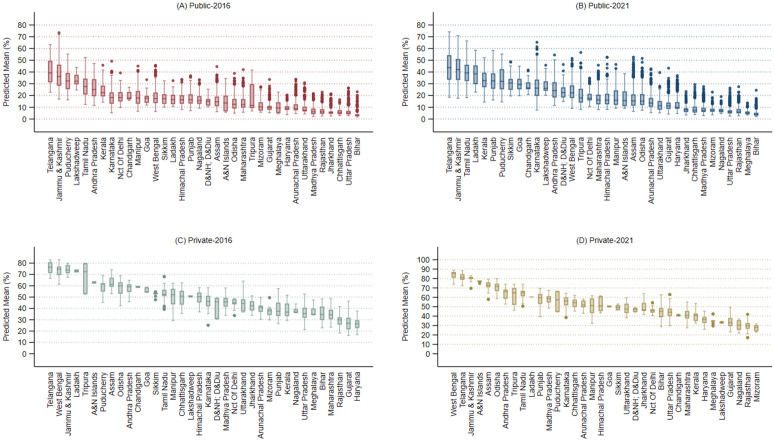
Box plots for cluster-level predictions (%) for cesarean section births by states, India, NFHS 2016 & 2021.

Fig 3 presents cluster-level predicted mean values of cesarean births for the public and private sectors in 2016 and 2021. Telangana ranked highest in predicted prevalence for the public sector in both years (Fig 3A and Fig 3B) and the private sector in 2016 (Fig 3C), while West Bengal ranked highest for private-sector births in 2021. Jammu and Kashmir, along with southern states such as Tamil Nadu, Kerala, and Andhra Pradesh, consistently ranked high across all four combinations, whereas northern, socioeconomically disadvantaged states like Bihar, Uttar Pradesh, Rajasthan, and Haryana ranked consistently low. Comparing across sectors, the predicted prevalence of cesarean births was notably high in the private sector of West Bengal, regardless of the year.

**Fig 4:**
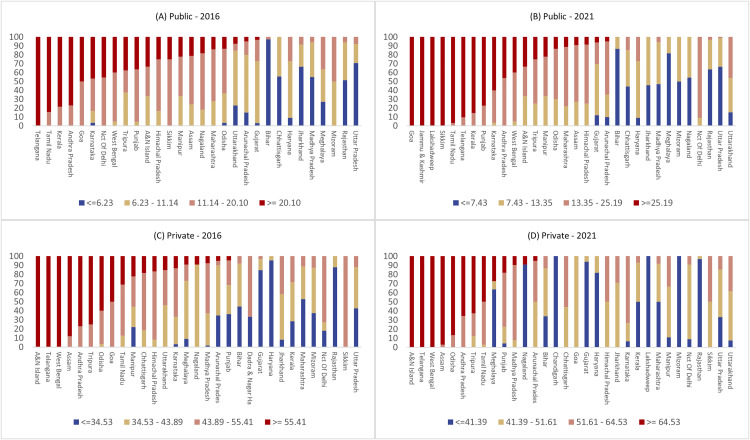
Distribution (%) of districts by prevalence of caesarean section births in public and private facilities across states, India, NFHS 2016 & 2021.

Fig 4 presents the distribution of districts by the prevalence of cesarean births in both public and private facilities across states. In the public sector, there was a noticeable increase in the number of states with only high-prevalence districts, indicating an overuse of cesarean sections. While in 2016, only Telangana had all of its districts classified as high prevalence, by 2021, other states such as Goa, Jammu and Kashmir, Lakshadweep, and Sikkim had reached this status. Additionally, states like Tamil Nadu, Telangana, and Kerala came close to having all their districts in the high-prevalence category. A similar trend was observed in the private sector, though the shift was more gradual and subtle. In contrast, states with a high number of low-prevalence districts, indicating underuse or limited access to cesarean sections, included Gujarat and Haryana, which ranked high in the private sector but had very low prevalence in the public sector. Conversely, states like Bihar and Uttar Pradesh, where the prevalence of cesarean sections was low in the private sector, ranked higher in the public sector, highlighting the disparity in access and availability of appropriate cesarean services between sectors. More interestingly, states like Jharkhand and Madhya Pradesh are showing a pattern of decrease in the number of low-prevalence districts for public hospitals, indicating improvement in the obstetric services and access to maternal healthcare. For example, Jharkhand had 64.7% of low-prevalence districts in 2016, which reduced to 45.8% in 2021. These patterns are also visible through maps reflecting change between 2016 and 2021 for both public and private facilities (see Fig B in S1 text for details).

**Fig 5:**
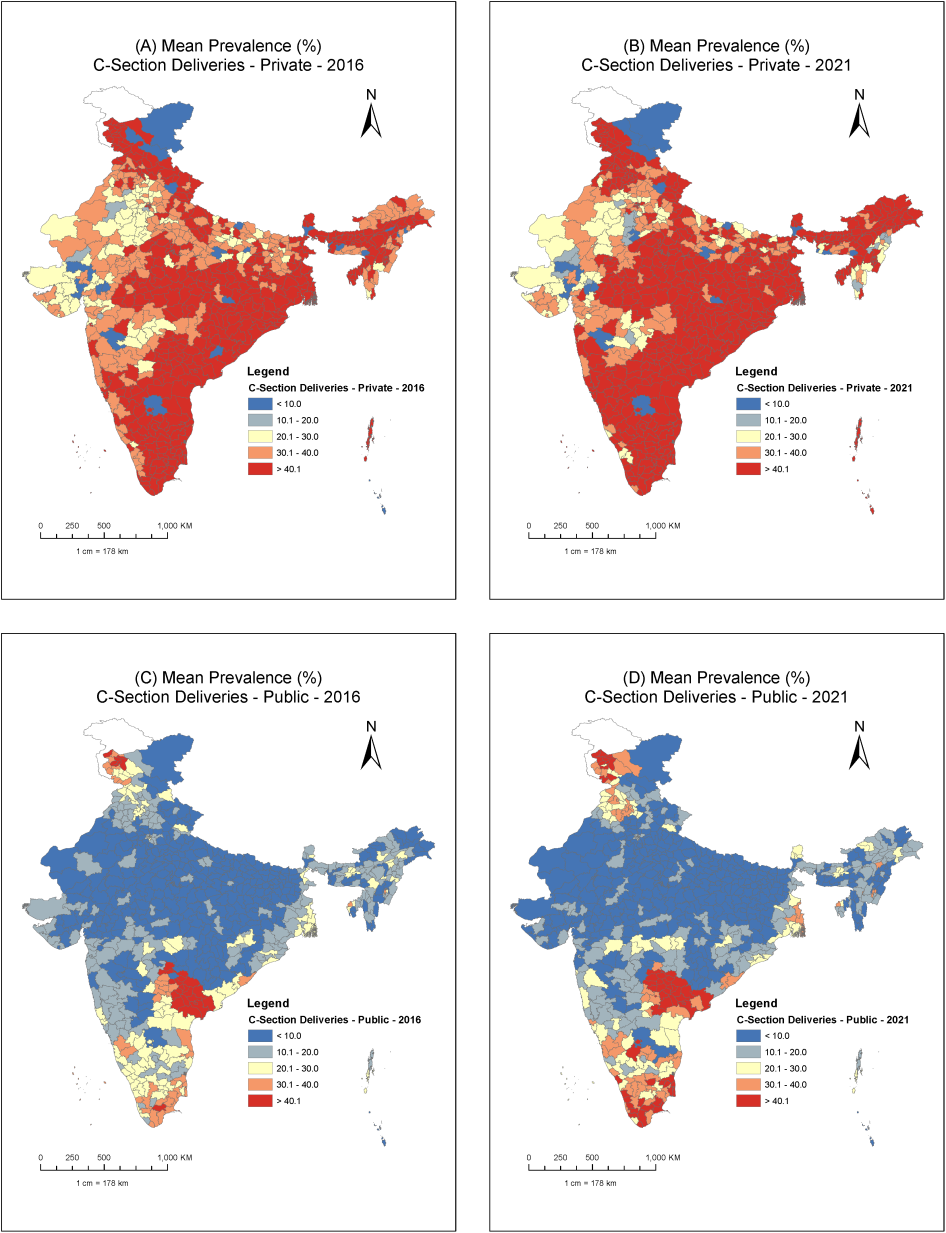
Maps for district-level predicted mean for cesarean section births across public and private facilities, India, NFHS 2016 & 2021.

Maps illustrating the district-level predicted mean for cesarean section births across public and private facilities (Fig 5) further confirm the notably higher prevalence (40.1% and above) of cesarean births in the private sector compared to the public sector. In the private sector, there was a visible increase in the number of high-prevalence districts, particularly in the southwest and northeast regions of the country. The clustering of high-prevalence districts was predominantly observed in the southern and eastern regions. Murshidabad (92.4%) and Nadia (92.3%) from West Bengal, and Karimnagar (91.9%) from Telangana exhibited the highest rates in 2021 (Table B in S1 Text). In contrast, low-prevalence districts were mostly concentrated in the northern, western, and central regions. Karauli (9.7%) in Rajasthan, Panch Mahals (14.8%) in Gujarat and Mokokchung (15.2%) in Nagaland exhibited the lowest rates in 2021. In the public sector, the majority of districts exhibited low-prevalence rates (below 10%), with these districts primarily clustered across the northern and central regions. High prevalence districts were concentrated within the states of Telangana, Jammu and Kashmir and Tamil Nadu, with a slight increase in their number in 2021. Jangaon (69.8%), Jagital (66.9%), Karimnagar and (66.6%) in Telangana, Pulwama (59.1%), Anantnag (56.5%), and Srinagar (53.1%) in Jammu and Kashmir, and Tirunelveli (54.8%), Kanniyakumari (52.2%), and Ariyalur (48.6%) in Tamil Nadu were the high prevalence districts for public sector in 2021 (Table B in S1 Text).

## Discussion

This study identified 4 major findings. First, at the national level between 2016 and 2021, the prevalence of births delivered by cesarean section slightly decreased in the public sector by 1.2% while it rose by 2.1% in the private sector. Interestingly, for public hospitals, even in the low-prevalence states, the prevalence has decreased (or is almost stagnant), indicating a need to strengthen public facilities for at least necessary cesarean births. Second, across all three geographies, interstate variation in cesarean section prevalence was the highest and has further increased over time. Third, districts that exhibited higher cesarean section prevalence in 2016 continued to show higher prevalence in 2021, indicating lesser improvements in high-prevalence districts. Fourth, within the southern states of Tamil Nadu, Telangana, and Kerala, the public sector exhibited high prevalence rates across most districts, whereas in the private sector, states such as Telangana, West Bengal, Assam, and Andhra Pradesh were predominantly characterized by districts with high cesarean section rates. A significant trend observed between 2016 and 2021 was the decrease in cesarean section deliveries within the public sector and an increase in the private sector. Telangana and Jammu and Kashmir were consistently flagged for their exceptionally high cesarean section rates in both sectors. In contrast, northern states such as Bihar, Jharkhand, and Uttar Pradesh reported the lowest prevalence in public hospitals, while Rajasthan maintained the lowest rates in private facilities. Over this period, disparities between states widened, particularly in the private sector.

The public-private disparities in cesarean section have been reported in several studies across India [19–21]. The substantial increase in cesarean section prevalence within private healthcare facilities, which is almost double compared to public facilities, may reflect mothers’ preference for a less stressful delivery process and the desire to schedule births at auspicious times [22]. The rise in private sector cesarean sections was attributed to several other factors, such as better facilities managing high-risk deliveries, shortages of specialists in public hospitals, and expanded insurance coverage through Ayushman Bharat, which encouraged more surgical interventions [23]. These findings underscore the need for policy interventions aimed at strengthening public health facilities, particularly emergency obstetric care services. Additionally, the “*Pradhan Mantri Surakshit Matritva Abhiyan*” (PMSMA) program was identified as a potential tool to mitigate service provider-induced increases in cesarean section rates within the private sector [21]. By leveraging PMSMA, state governments could implement standardized protocols, enhance monitoring, and regulate private healthcare practices to ensure that cesarean sections are performed based on medical necessity, thereby reducing unnecessary surgical interventions and promoting equitable maternal health outcomes across states. Additionally, enhancing the capacity and quality of public hospitals was essential to ensure that medically necessary cesarean sections could be performed within the public sector, thereby reducing reliance on profit-driven private healthcare facilities and addressing the growing disparities in cesarean section prevalence across states [20].

In contrast to other studies focusing on health outcomes such as mild and moderate anemia among women [24] and child under-nutrition [25], which reported high-level variation in the cluster level and district level, respectively, this study revealed that state-level variation in cesarean section rates was predominant. This interstate variation was the highest among all geographic levels and was found to have increased over the study period. States like Telangana, Jammu and Kashmir, and Tamil Nadu consistently exhibited exceptionally high cesarean section rates, while northern states such as Bihar, Jharkhand, Uttar Pradesh, and Rajasthan were identified for their low prevalence rates. The elevated interstate disparities are reflective of a comparatively higher prevalence of institutional births (especially in the private sector) [26] in southern states, which is critical since the likelihood of C-section is significantly greater in private facilities. Sociological factors such as female education, late child-rearing, and urbanization could also explain the higher cesarean section rates. [27–30]. Substantial state-level heterogeneities in cesarean section rates give rise to the argument that government policies should prioritize inter-state variation when designing targeting strategies. Strengthening state-specific healthcare infrastructure, enhancing provider training, and implementing standardized guidelines were recommended to address the growing inconsistencies in obstetric care across different regions. Focusing on state-level policies will mitigate the entrenched disparities and promote equitable and evidence-based maternal healthcare practices nationwide. The findings from this study can be used by maternal healthcare-focused programs to determine the appropriate geographic/policy unit for efficiently channeling efforts and resources.

A strong correlation was observed between district-level cesarean section prevalence in 2016 and 2021 across both public and private sectors. Districts with high cesarean section rates in 2016 continued to maintain elevated rates in 2021, indicating that existing interventions were insufficient to reduce unnecessary procedures or to elevate rates in low-prevalence areas. This persistence underscored the need for targeted strategies to address inequalities in cesarean section practices. Disparities were linked to variations in healthcare service quality, accessibility, and socioeconomic conditions [31]. It was noted that many healthcare centers lacked equitable distribution of quality human resources, effective communication systems, adherence to institutional delivery standards, and essential obstetric care facilities. Furthermore, districts characterized by lower socioeconomic levels, extreme poverty, and marginalization required prioritization for additional funding and effective management [32]. Addressing these inequalities necessitated policy interventions focused on strengthening public healthcare infrastructure, ensuring equitable distribution of healthcare resources, and allocating financial support to underdeveloped and marginalized districts.

Lastly, a significant increase in the number of states characterized by districts with high prevalence has occurred between 2016 and 2021. The distribution analysis revealed that the number of states with exclusively high prevalence districts increased significantly in the public sector, expanding from only Telangana in 2016 to include Goa, Jammu and Kashmir, Lakshadweep, and Sikkim by 2021. This finding is replicated in other studies, highlighting a rise in cesarean section deliveries in southern states due to factors ranging from lesser pain tolerance [33] to higher levels of education received by the women [34]. This strengthens the argument that the overall rise in cesarean section deliveries across most districts underscores the need for targeted government interventions aimed at reducing unnecessary surgical births. Most of the policy efforts in India are indirect – via improving access to timely antenatal and maternal healthcare – to reduce the burden of c-section births. However, states like Karnataka and Andhra Pradesh have implemented C-section audits and behavioral programs to promote natural deliveries. Policy measures should focus on standardizing obstetric practices, enhancing training for healthcare providers, and regulating cesarean section rates to ensure that deliveries are performed based on medical necessity rather than regional or economic incentives. Preventing unnecessary cesarean sections should be a key focus of continuing education programs for medical officers and obstetricians [35]. In the coming years, India must work towards achieving nearly universal access to institutional deliveries and maintain cesarean section rates at optimal levels. While the optimal levels recommended by WHO guidelines in 1985 were maintaining national average rates between 10% and 15% [7]. However, this threshold was later withdrawn to focus on medical necessity rather than numerical targets [36]. Regardless, the global concern around rising cesarean sections remains valid. In the absence of clinical indications, there is no evidence to suggest that cesarean methods provide additional benefits, and rates exceeding 10% have not been associated with reductions in maternal or neonatal mortality [36]. Further, normative thresholds for optimal prevalence of cesarean sections should have context-specific empirical backing. A universal threshold for all countries (geographies, socioeconomic groups) to follow may be worth reconsidering, and hence, there is a clear scope for research investment in the subject matter. Although previous studies have identified differential thresholds for c-section deliveries based on Robson’s classification, further research on universalizing these cut-offs in a diverse country like India warrants large-scale investment [37–39].

The following are the limitations of this study. Firstly, the cross-sectional nature of the data restricts us from performing a comparative analysis over a longer period of time for the same households. Second, it was difficult to obtain statistically robust estimates for some of the States and Union Territories as the sample size was very small. Hence, we presented the broad trends and patterns without adjusting for the socioeconomic characteristics. Third, NFHS has used the sampling frame of the 2011 Census for sampling. The data is available for 640 districts for 2016. However, in 2021, there were more than 700 districts. However, we believe that the same trends in multi-level variation will remain. Additionally, the lowest geographical administrative unit was a cluster (PSU).

## Supporting information

S1 Text Supplementary Exhibits. (DOCX)
