# Supplementary material for: Trends in nulliparous singleton alive births by cesarean section in India: Empirical patterns across public and private hospitals for 720 districts, 2016–2021
Source: PLOS Glob Public Health. 2025 Nov 25;5(11):e0005501. doi: 10.1371/journal.pgph.0005501 (PMC12646418; doi:10.1371/journal.pgph.0005501)
Supplement: S1 Text — (DOCX) [file pgph.0005501.s001.docx]

**Supplementary to:**

**Trends in nulliparous singleton alive births by cesarean section in India: Empirical patterns across public and private hospitals for 720 districts, 2016-2021**

**Supporting Information**

**Fig A. Flow diagram showing exclusions and final sample sizes for primary analysis of the study** **population, India, NFHS 2016 & 2021**

Original Sample Size (Kids Recode)

| NFHS 4 | NFHS 5 |
| --- | --- |
| 259,627 | 232920 |

Exclusion – Dead Children

| NFHS 4 | NFHS 5 |
| --- | --- |
| 11884 | 8702 |

Alive Children

| NFHS 4 | NFHS 5 |
| --- | --- |
| 247743 | 224218 |

Exclusion – Indicator Specific

| Component | NFHS 4 | NFHS 5 |
| --- | --- | --- |
| Birth Order >1 | 155871 | 138390 |
| Twins | 628 | 653 |

Final Analytic Sample – Nulliparous with a Term Singleton Alive Births in Last Five Years

| NFHS 4 | NFHS 5 |
| --- | --- |
| **91244** | **85175** |

**Table A. Distribution (%) of cesarean section deliveries across public and private facilities by states, India, NFHS 2016 & 2021**

|  | 2016 | | 2021 | |
| --- | --- | --- | --- | --- |
| State | Public (%) | Private (%) | Public (%) | Private (%) |
| Andhra Pradesh | 21.6 | 76.4 | 31.5 | 67.8 |
| Arunachal Pradesh | 60.9 | 38.7 | 86.1 | 13.8 |
| Assam | 56.4 | 43.6 | 62.3 | 37.4 |
| Bihar | 20.2 | 78.5 | 21.2 | 77.2 |
| Chhattisgarh | 31.6 | 66.3 | 41.1 | 58.5 |
| Delhi | 61.6 | 38.4 | 46.8 | 52.9 |
| Goa | 26.6 | 73.4 | 44.8 | 55.2 |
| Gujarat | 19.5 | 75.8 | 25.4 | 70.6 |
| Haryana | 37.2 | 60.9 | 34.6 | 65.0 |
| Himachal Pradesh | 65.1 | 34.7 | 59.6 | 40.2 |
| Jharkhand | 21.4 | 76.4 | 30.9 | 67.5 |
| Karnataka | 39.7 | 59.6 | 46.3 | 53.1 |
| Kerala | 27.3 | 72.4 | 32.6 | 67.1 |
| Madhya Pradesh | 45.9 | 52.2 | 54.7 | 44.9 |
| Maharashtra | 28.7 | 69.7 | 40.1 | 57.9 |
| Manipur | 49.6 | 50.1 | 57.3 | 42.1 |
| Meghalaya | 46.2 | 53.8 | 55.3 | 44.6 |
| Mizoram | 65.9 | 22.8 | 66.4 | 31.7 |
| Nagaland | 62.3 | 37.7 | 55.1 | 44.9 |
| Odisha | 64.2 | 35.4 | 55.7 | 43.9 |
| Punjab | 35.8 | 63.5 | 41.7 | 58.1 |
| Rajasthan | 41.5 | 58.5 | 53.7 | 46.2 |
| Sikkim | 68.4 | 31.6 | 72.8 | 27.2 |
| Tamil Nadu | 48.0 | 51.4 | 53.6 | 46.3 |
| Telangana | 18.2 | 81.3 | 36.4 | 63.1 |
| Tripura | 63.2 | 36.2 | 71.2 | 28.8 |
| Uttar Pradesh | 21.9 | 77.1 | 26.0 | 72.8 |
| Uttarakhand | 28.8 | 69.2 | 36.6 | 62.6 |
| West Bengal | 41.7 | 57.6 | 50.8 | 49.1 |
| All India | 34.8 | 64.0 | 41.2 | 58.0 |

**Table B. Distribution (%) of cesarean section deliveries across public and private facilities by districts, India, NFHS 2016 & 2021**

| **Sr. No.** | **District** | **Public** | | **Private** | |
| --- | --- | --- | --- | --- | --- |
|  |  | **2016** | **2021** | **2016** | **2021** |
| 1 | Alluri Sitharama Raju | 20.3 | 16.4 | 61.8 | 65.1 |
| 2 | Anakapalli | 22.0 | 20.8 | 61.8 | 60.1 |
| 3 | Ananthapuramu | 16.6 | 16.7 | 60.0 | 51.0 |
| 4 | Annamayya | 18.6 | 20.5 | 49.8 | 51.9 |
| 5 | Bapatla | 27.8 | 24.8 | 57.4 | 70.7 |
| 6 | Chittoor | 23.3 | 9.8 | 54.4 | 54.9 |
| 7 | Dr. B.R. Ambedkar Konaseema | 19.2 | 36.2 | 59.7 | 69.8 |
| 8 | East Godavari | 28.3 | 42.2 | 60.9 | 77.4 |
| 9 | Eluru | 42.5 | 36.5 | 63.5 | 68.0 |
| 10 | Guntur | 40.4 | 34.7 | 61.3 | 75.8 |
| 11 | Kakinada | 18.4 | 29.8 | 59.7 | 72.8 |
| 12 | Krishna | 40.8 | 45.6 | 64.8 | 79.1 |
| 13 | Kurnool | 22.5 | 16.1 | 54.4 | 67.5 |
| 14 | Nandyal | 22.9 | 17.1 | 54.4 | 59.2 |
| 15 | Ntr | 34.8 | 42.8 | 64.8 | 78.2 |
| 16 | Palnadu | 36.0 | 31.6 | 61.3 | 70.3 |
| 17 | Parvathipuram Manyam | 30.2 | 31.2 | 56.5 | 76.7 |
| 18 | Prakasam | 24.8 | 29.4 | 54.5 | 62.7 |
| 19 | Sri Potti Sriramulu Nellore | 37.7 | 20.4 | 58.0 | 60.9 |
| 20 | Sri Sathya Sai | 15.3 | 12.6 | 60.0 | 41.6 |
| 21 | Srikakulam | 35.9 | 37.5 | 59.1 | 74.2 |
| 22 | Tirupati | 28.3 | 16.7 | 57.1 | 62.5 |
| 23 | Visakhapatnam | 20.6 | 17.7 | 61.3 | 57.3 |
| 24 | Vizianagaram | 26.3 | 30.4 | 55.8 | 69.4 |
| 25 | West Godavari | 41.9 | 38.7 | 63.1 | 73.4 |
| 26 | Y.S.R. | 14.7 | 25.7 | 45.9 | 47.7 |
| 27 | Kolkata | 36.3 | 28.2 | 76.1 | 85.0 |
| 28 | Kupwara | 44.2 | 49.0 | 71.1 | 83.6 |
| 29 | Badgam | 50.7 | 44.5 | 73.2 | 85.0 |
| 30 | Leh | 21.3 | 47.3 | 74.4 | 71.1 |
| 31 | Punch | 30.3 | 34.6 | 76.7 | 89.3 |
| 32 | Rajouri | 31.6 | 30.2 | 71.8 | 82.0 |
| 33 | Kathua | 30.4 | 29.9 | 74.7 | 84.4 |
| 34 | Baramula | 37.8 | 52.0 | 77.5 | 85.7 |
| 35 | Bandipore | 37.2 | 49.9 | 67.5 | 63.1 |
| 36 | Srinagar | 65.9 | 53.1 | 79.8 | 88.9 |
| 37 | Ganderbal | 53.6 | 49.7 | 74.8 | 89.0 |
| 38 | Pulwama | 56.9 | 59.1 | 78.5 | 90.9 |
| 39 | Shupiyan | 49.0 | 29.1 | 79.8 | 86.9 |
| 40 | Anantnag | 40.8 | 56.5 | 77.9 | 86.6 |
| 41 | Kulgam | 38.0 | 44.8 | 70.4 | 89.9 |
| 42 | Doda | 27.2 | 23.7 | 76.1 | 80.4 |
| 43 | Ramban | 25.9 | 20.9 | 73.7 | 80.3 |
| 44 | Kishtwar | 27.8 | 35.2 |  | 86.5 |
| 45 | Udhampur | 22.4 | 41.4 | 67.5 | 87.3 |
| 46 | Reasi | 28.1 | 32.6 | 73.9 | 67.3 |
| 47 | Jammu | 34.4 | 42.9 | 78.3 | 82.7 |
| 48 | Samba | 31.4 | 36.3 | 71.4 | 80.9 |
| 49 | Chamba | 10.1 | 8.0 | 58.3 | 56.0 |
| 50 | Kangra | 22.0 | 19.6 | 44.7 | 68.9 |
| 51 | Lahul & Spiti | 14.4 | 14.4 | 55.7 | 49.2 |
| 52 | Kullu | 9.3 | 9.0 | 52.7 | 42.0 |
| 53 | Mandi | 13.8 | 11.4 | 45.3 | 46.7 |
| 54 | Hamirpur | 19.2 | 13.0 | 48.8 | 44.4 |
| 55 | Una | 17.6 | 29.6 | 54.0 | 56.2 |
| 56 | Bilaspur | 14.6 | 16.2 | 37.0 | 32.1 |
| 57 | Solan | 21.6 | 20.3 | 53.9 | 45.5 |
| 58 | Sirmaur | 18.2 | 8.5 | 47.4 | 54.6 |
| 59 | Shimla | 16.1 | 13.6 | 45.5 | 31.6 |
| 60 | Kinnaur | 24.0 | 16.1 | 52.1 | 59.5 |
| 61 | Kapurthala | 20.2 | 32.2 | 38.7 | 62.0 |
| 62 | Jalandhar | 13.8 | 39.6 | 46.9 | 60.8 |
| 63 | Hoshiarpur | 20.9 | 22.2 | 37.6 | 63.9 |
| 64 | Shahid Bhagat Singh Nagar | 23.9 | 20.6 | 41.7 | 59.6 |
| 65 | Fatehgarh Sahib | 27.2 | 32.4 | 39.4 | 57.3 |
| 66 | Ludhiana | 11.6 | 21.2 | 32.0 | 61.1 |
| 67 | Moga | 18.4 | 25.4 | 33.3 | 37.9 |
| 68 | Muktsar | 8.8 | 15.1 | 26.7 | 27.0 |
| 69 | Faridkot | 15.9 | 23.0 | 34.9 | 37.7 |
| 70 | Bathinda | 15.1 | 25.9 | 40.6 | 47.2 |
| 71 | Mansa | 16.1 | 28.0 | 33.4 | 47.2 |
| 72 | Patiala | 23.9 | 39.3 | 31.8 | 54.3 |
| 73 | Amritsar | 11.9 | 26.9 | 45.1 | 60.7 |
| 74 | Tarn Taran | 15.7 | 21.3 | 36.1 | 58.6 |
| 75 | Rupnagar | 16.7 | 30.0 | 46.2 | 48.2 |
| 76 | Sahibzada Ajit Singh Nagar | 23.1 | 31.9 | 47.1 | 42.8 |
| 77 | Sangrur | 11.6 | 33.0 | 57.4 | 47.4 |
| 78 | Barnala | 16.9 | 32.8 | 32.7 | 51.3 |
| 79 | Chandigarh | 19.3 | 26.2 | 59.0 | 45.2 |
| 80 | Uttarkashi | 6.5 | 4.7 | 40.1 | 35.2 |
| 81 | Chamoli | 5.7 | 6.2 | 42.0 | 54.8 |
| 82 | Rudraprayag | 10.9 | 13.2 | 50.8 | 51.8 |
| 83 | Tehri Garhwal | 4.8 | 7.9 | 44.4 | 43.4 |
| 84 | Dehradun | 7.4 | 18.5 | 37.3 | 36.8 |
| 85 | Garhwal | 6.9 | 5.8 | 58.5 | 38.6 |
| 86 | Pithoragarh | 5.8 | 14.4 | 43.4 | 44.4 |
| 87 | Bageshwar | 10.0 | 8.2 | 39.6 | 61.2 |
| 88 | Almora | 7.6 | 12.0 | 46.8 | 58.9 |
| 89 | Champawat | 7.4 | 6.1 | 50.3 | 46.8 |
| 90 | Nainital | 21.9 | 20.6 | 64.0 | 64.8 |
| 91 | Udham Singh Nagar | 10.2 | 5.6 | 48.1 | 47.3 |
| 92 | Hardwar | 12.5 | 14.0 | 36.8 | 36.4 |
| 93 | Panchkula | 11.3 | 27.6 | 22.6 | 40.9 |
| 94 | Ambala | 11.0 | 23.1 | 22.4 | 41.6 |
| 95 | Yamunanagar | 11.3 | 22.1 | 23.8 | 32.0 |
| 96 | Kurukshetra | 11.7 | 9.8 | 32.3 | 39.4 |
| 97 | Kaithal | 9.4 | 10.0 | 28.1 | 49.6 |
| 98 | Karnal | 11.7 | 5.6 | 23.9 | 38.0 |
| 99 | Panipat | 5.6 | 8.2 | 31.3 | 31.8 |
| 100 | Sonipat | 8.3 | 6.7 | 23.4 | 29.8 |
| 101 | Jind | 4.1 | 8.3 | 24.1 | 24.2 |
| 102 | Fatehabad | 10.2 | 11.1 | 17.1 | 37.1 |
| 103 | Sirsa | 13.9 | 12.6 | 37.6 | 47.2 |
| 104 | Hisar | 10.2 | 10.1 | 26.4 | 26.0 |
| 105 | Rohtak | 9.8 | 9.4 | 30.2 | 35.1 |
| 106 | Jhajjar | 8.5 | 8.7 | 26.8 | 32.5 |
| 107 | Mahendragarh | 7.4 | 12.7 | 28.4 | 35.5 |
| 108 | Rewari | 8.1 | 9.2 | 27.6 | 42.9 |
| 109 | Gurgaon | 7.8 | 7.0 | 16.8 | 34.0 |
| 110 | Mewat | 8.0 | 3.2 | 29.5 | 16.6 |
| 111 | Faridabad | 12.4 | 6.5 | 30.4 | 36.5 |
| 112 | Palwal | 9.3 | 5.8 | 22.6 | 25.7 |
| 113 | Ganganagar | 9.6 | 6.3 | 35.2 | 33.5 |
| 114 | Hanumangarh | 5.6 | 7.0 | 28.1 | 34.6 |
| 115 | Bikaner | 8.8 | 5.4 | 34.9 | 32.2 |
| 116 | Churu | 3.8 | 3.7 | 18.3 | 23.8 |
| 117 | Jhunjhunun | 4.0 | 4.5 | 20.9 | 27.6 |
| 118 | Alwar | 4.6 | 5.2 | 28.4 | 18.8 |
| 119 | Bharatpur | 6.5 | 5.7 | 30.2 | 38.4 |
| 120 | Dhaulpur | 4.0 | 3.9 | 36.4 | 26.0 |
| 121 | Karauli | 3.0 | 2.1 | 21.2 | 9.7 |
| 122 | Sawai Madhopur | 3.9 | 7.4 | 20.1 | 18.0 |
| 123 | Dausa | 7.8 | 4.7 | 27.4 | 19.3 |
| 124 | Jaipur | 15.3 | 14.6 | 30.0 | 27.2 |
| 125 | Sikar | 6.8 | 9.9 | 27.4 | 27.1 |
| 126 | Nagaur | 6.1 | 5.0 | 25.4 | 23.0 |
| 127 | Jodhpur | 12.8 | 7.6 | 30.8 | 31.0 |
| 128 | Jaisalmer | 4.4 | 7.9 | 28.9 | 25.4 |
| 129 | Barmer | 3.3 | 3.3 | 32.2 | 21.1 |
| 130 | Jalor | 3.1 | 1.8 | 19.0 | 15.4 |
| 131 | Sirohi | 4.0 | 5.3 | 22.7 | 17.7 |
| 132 | Pali | 7.9 | 3.7 | 31.5 | 36.1 |
| 133 | Ajmer | 9.1 | 7.8 | 32.1 | 24.9 |
| 134 | Tonk | 5.3 | 7.1 | 26.6 | 32.6 |
| 135 | Bundi | 9.1 | 6.5 | 41.6 | 32.7 |
| 136 | Bhilwara | 4.8 | 3.3 | 27.7 | 27.6 |
| 137 | Rajsamand | 7.0 | 4.3 | 25.6 | 37.4 |
| 138 | Dungarpur | 5.0 | 3.6 | 31.8 | 22.1 |
| 139 | Banswara | 7.7 | 4.0 | 29.2 | 20.3 |
| 140 | Chittaurgarh | 6.2 | 8.8 | 33.2 | 45.8 |
| 141 | Kota | 6.2 | 13.1 | 33.3 | 35.3 |
| 142 | Baran | 6.4 | 3.7 | 33.5 | 32.0 |
| 143 | Jhalawar | 8.9 | 5.1 | 32.6 | 30.1 |
| 144 | Udaipur | 9.9 | 4.5 | 32.0 | 33.2 |
| 145 | Pratapgarh | 2.8 | 1.9 | 34.0 | 34.4 |
| 146 | Saharanpur | 19.4 | 7.0 | 32.5 | 37.7 |
| 147 | Bijnor | 4.9 | 3.2 | 34.9 | 39.8 |
| 148 | Rampur | 10.4 | 3.8 | 37.9 | 39.0 |
| 149 | Jyotiba Phule Nagar | 4.2 | 4.9 | 35.2 | 36.6 |
| 150 | Meerut | 8.2 | 9.3 | 35.4 | 28.8 |
| 151 | Baghpat | 3.8 | 5.8 | 29.2 | 24.1 |
| 152 | Gautam Buddha Nagar | 12.1 | 5.1 | 23.2 | 29.3 |
| 153 | Bulandshahr | 3.4 | 4.7 | 31.9 | 37.8 |
| 154 | Aligarh | 7.9 | 4.9 | 44.0 | 43.9 |
| 155 | Mahamaya Nagar | 3.5 | 4.3 | 29.9 | 40.8 |
| 156 | Mathura | 5.1 | 3.7 | 30.9 | 33.8 |
| 157 | Agra | 7.4 | 6.9 | 35.9 | 33.2 |
| 158 | Firozabad | 5.5 | 2.2 | 37.9 | 39.2 |
| 159 | Mainpuri | 3.6 | 2.1 | 30.8 | 31.0 |
| 160 | Bareilly | 8.7 | 4.2 | 40.8 | 38.0 |
| 161 | Pilibhit | 3.0 | 6.4 | 39.8 | 48.6 |
| 162 | Shahjahanpur | 5.1 | 3.7 | 40.5 | 41.4 |
| 163 | Sitapur | 5.9 | 3.6 | 33.1 | 36.8 |
| 164 | Hardoi | 7.6 | 2.2 | 42.9 | 42.3 |
| 165 | Unnao | 6.4 | 7.4 | 40.6 | 51.5 |
| 166 | Lucknow | 14.3 | 18.5 | 47.7 | 53.0 |
| 167 | Farrukhabad | 6.5 | 3.2 | 35.0 | 39.0 |
| 168 | Kannauj | 3.2 | 3.2 | 32.7 | 47.1 |
| 169 | Etawah | 5.4 | 4.9 | 33.3 | 44.9 |
| 170 | Auraiya | 4.2 | 2.0 | 34.0 | 34.1 |
| 171 | Kanpur Dehat | 6.3 | 1.9 | 34.6 | 42.4 |
| 172 | Kanpur Nagar | 6.1 | 10.0 | 39.5 | 45.1 |
| 173 | Jalaun | 4.2 | 3.2 | 34.0 | 55.0 |
| 174 | Jhansi | 6.1 | 4.9 | 48.4 | 47.5 |
| 175 | Lalitpur | 3.0 | 4.1 | 29.5 | 46.7 |
| 176 | Hamirpur | 5.3 | 5.1 | 36.6 | 51.4 |
| 177 | Mahoba | 3.3 | 3.9 | 37.5 | 55.6 |
| 178 | Banda | 4.4 | 2.1 | 31.3 | 49.1 |
| 179 | Chitrakoot | 3.8 | 2.4 | 36.1 | 46.4 |
| 180 | Fatehpur | 3.5 | 3.0 | 35.9 | 31.0 |
| 181 | Pratapgarh | 5.0 | 4.0 | 34.7 | 29.2 |
| 182 | Kaushambi | 3.8 | 2.9 | 37.0 | 39.3 |
| 183 | Allahabad | 5.8 | 3.1 | 39.3 | 43.7 |
| 184 | Bara Banki | 11.5 | 6.3 | 52.6 | 30.9 |
| 185 | Faizabad | 4.6 | 6.6 | 34.1 | 56.6 |
| 186 | Ambedkar Nagar | 3.1 | 5.3 | 25.2 | 29.5 |
| 187 | Bahraich | 5.1 | 3.8 | 43.7 | 27.7 |
| 188 | Shrawasti | 4.0 | 2.0 | 24.3 | 30.7 |
| 189 | Balrampur | 8.0 | 6.6 | 51.2 | 51.1 |
| 190 | Gonda | 5.7 | 5.4 | 37.8 | 39.1 |
| 191 | Siddharthnagar | 3.1 | 3.0 | 32.4 | 26.0 |
| 192 | Basti | 4.3 | 4.3 | 40.6 | 50.1 |
| 193 | Sant Kabir Nagar | 5.2 | 4.3 | 27.5 | 33.3 |
| 194 | Maharajganj | 7.2 | 5.4 | 49.8 | 53.7 |
| 195 | Gorakhpur | 7.8 | 4.6 | 48.1 | 58.2 |
| 196 | Kushinagar | 5.3 | 2.9 | 36.0 | 48.5 |
| 197 | Deoria | 4.0 | 3.7 | 37.1 | 49.3 |
| 198 | Azamgarh | 3.9 | 5.3 | 27.3 | 28.6 |
| 199 | Mau | 3.8 | 3.4 | 28.4 | 35.5 |
| 200 | Ballia | 3.3 | 2.5 | 30.6 | 34.2 |
| 201 | Jaunpur | 5.4 | 2.4 | 35.1 | 35.0 |
| 202 | Ghazipur | 5.0 | 3.7 | 31.6 | 44.4 |
| 203 | Chandauli | 3.9 | 4.0 | 47.3 | 45.1 |
| 204 | Varanasi | 10.3 | 6.9 | 45.1 | 44.1 |
| 205 | Sant Ravidas Nagar | 5.4 | 4.6 | 21.3 | 38.9 |
| 206 | Mirzapur | 6.3 | 5.8 | 23.6 | 40.0 |
| 207 | Sonbhadra | 6.3 | 3.0 | 40.2 | 43.7 |
| 208 | Etah | 4.0 | 2.3 | 27.1 | 25.9 |
| 209 | Kanshiram Nagar | 4.1 | 1.9 | 33.5 | 20.3 |
| 210 | Pashchim Champaran | 2.6 | 2.1 | 34.6 | 28.7 |
| 211 | Purba Champaran | 4.2 | 2.0 | 25.7 | 23.4 |
| 212 | Sheohar | 3.1 | 1.8 | 35.3 | 38.8 |
| 213 | Sitamarhi | 3.2 | 3.6 | 34.6 | 39.8 |
| 214 | Madhubani | 4.2 | 2.8 | 38.7 | 49.9 |
| 215 | Supaul | 2.6 | 1.9 | 32.1 | 29.5 |
| 216 | Araria | 3.1 | 3.3 | 42.9 | 43.6 |
| 217 | Kishanganj | 13.3 | 4.8 | 48.3 | 66.2 |
| 218 | Purnia | 2.4 | 2.5 | 34.3 | 37.7 |
| 219 | Katihar | 2.4 | 1.9 | 40.2 | 39.0 |
| 220 | Madhepura | 2.0 | 1.0 | 39.1 | 40.7 |
| 221 | Saharsa | 1.7 | 2.8 | 42.2 | 42.3 |
| 222 | Darbhanga | 5.9 | 3.9 | 37.7 | 49.0 |
| 223 | Muzaffarpur | 3.9 | 2.8 | 37.8 | 44.1 |
| 224 | Gopalganj | 3.2 | 2.5 | 34.3 | 31.8 |
| 225 | Siwan | 3.8 | 3.5 | 22.9 | 32.7 |
| 226 | Saran | 6.0 | 1.8 | 43.9 | 42.3 |
| 227 | Vaishali | 4.1 | 2.6 | 29.8 | 38.0 |
| 228 | Samastipur | 2.3 | 1.1 | 35.6 | 44.6 |
| 229 | Begusarai | 2.7 | 1.1 | 36.4 | 23.7 |
| 230 | Khagaria | 2.4 | 1.0 | 27.7 | 31.8 |
| 231 | Bhagalpur | 3.1 | 3.7 | 33.0 | 31.6 |
| 232 | Banka | 3.5 | 3.7 | 27.9 | 40.3 |
| 233 | Munger | 2.3 | 5.6 | 31.0 | 44.7 |
| 234 | Lakhisarai | 2.6 | 2.3 | 30.6 | 43.3 |
| 235 | Sheikhpura | 2.9 | 6.0 | 27.4 | 46.1 |
| 236 | Nalanda | 3.6 | 6.5 | 35.9 | 45.6 |
| 237 | Patna | 4.8 | 5.2 | 41.8 | 46.8 |
| 238 | Bhojpur | 3.9 | 2.8 | 32.1 | 35.7 |
| 239 | Buxer | 2.4 | 1.0 | 29.0 | 23.6 |
| 240 | Kaimur (Bhabua) | 2.2 | 1.4 | 26.0 | 32.0 |
| 241 | Rohtas | 3.8 | 1.6 | 28.0 | 33.8 |
| 242 | Gaya | 4.5 | 1.8 | 42.5 | 48.4 |
| 243 | Nawada | 2.7 | 1.2 | 37.2 | 42.5 |
| 244 | Jamui | 4.0 | 2.0 | 31.9 | 31.1 |
| 245 | Jehanabad | 3.4 | 2.7 | 42.2 | 63.3 |
| 246 | Arwal | 4.4 | 1.4 | 47.7 | 55.1 |
| 247 | North District | 18.0 | 28.0 | 50.8 | 55.0 |
| 248 | West District | 12.4 | 22.9 | 47.7 | 54.7 |
| 249 | South District | 13.6 | 25.4 | 54.7 | 63.5 |
| 250 | East District | 23.1 | 29.1 | 53.2 | 51.1 |
| 251 | Tawang | 13.4 | 17.0 | 42.5 | 64.3 |
| 252 | West Kameng | 9.4 | 9.3 | 33.8 | 50.5 |
| 253 | East Kameng | 8.9 | 9.8 | 31.2 | 52.6 |
| 254 | Papum Pare | 22.5 | 19.0 | 42.5 | 37.6 |
| 255 | Upper Subansiri | 14.5 | 18.1 | 34.0 | 48.9 |
| 256 | Upper Siang | 9.7 | 17.5 | 40.1 | 53.9 |
| 257 | Changlang | 7.0 | 12.7 | 49.6 | 54.4 |
| 258 | Lower Subansiri | 10.4 | 14.9 | 38.1 | 59.0 |
| 259 | Dibang Valley | 9.6 | 7.0 | 36.9 | 62.0 |
| 260 | Lower Dibang Valley | 6.1 | 8.3 | 38.7 | 53.6 |
| 261 | Anjaw | 8.5 | 14.2 | 39.7 | 54.3 |
| 262 | Mon | 11.2 | 3.4 | 38.5 | 33.5 |
| 263 | Mokokchung | 15.2 | 6.1 | 40.8 | 15.2 |
| 264 | Zunheboto | 19.0 | 12.0 | 41.7 | 35.9 |
| 265 | Wokha | 19.2 | 7.3 | 37.3 | 29.6 |
| 266 | Dimapur | 21.4 | 8.9 | 36.4 | 26.7 |
| 267 | Phek | 25.0 | 4.0 | 39.5 | 22.6 |
| 268 | Tuensang | 10.8 | 3.8 | 39.2 | 18.6 |
| 269 | Longleng | 10.6 | 6.6 |  | 17.5 |
| 270 | Kiphire | 12.0 | 3.9 | 41.9 | 28.6 |
| 271 | Kohima | 15.0 | 4.4 | 36.4 | 16.3 |
| 272 | Peren | 13.7 | 4.3 | 43.9 | 19.3 |
| 273 | Senapati | 8.2 | 9.2 | 49.4 | 48.5 |
| 274 | Tamenglong | 7.3 | 13.5 | 44.2 | 46.1 |
| 275 | Churachandpur | 8.1 | 10.1 | 29.3 | 26.8 |
| 276 | Bishnupur | 15.6 | 32.0 | 57.4 | 63.9 |
| 277 | Thoubal | 19.6 | 15.7 | 43.6 | 46.0 |
| 278 | Imphal West | 30.3 | 32.7 | 62.2 | 68.0 |
| 279 | Imphal East | 22.7 | 30.6 | 52.1 | 54.5 |
| 280 | Ukhrul | 18.6 | 6.6 | 33.5 | 24.1 |
| 281 | Chandel | 14.3 | 16.3 | 36.6 | 43.5 |
| 282 | Mamit | 6.8 | 3.4 | 34.2 | 27.5 |
| 283 | Kolasib | 11.4 | 5.9 | 31.7 | 27.1 |
| 284 | Aizawl | 18.4 | 10.4 | 41.1 | 31.8 |
| 285 | Champhai | 8.4 | 4.8 | 36.7 | 27.1 |
| 286 | Serchhip | 12.6 | 10.7 | 39.9 | 33.5 |
| 287 | Lunglei | 16.3 | 5.3 | 37.9 | 15.4 |
| 288 | Lawngtlai | 6.9 | 4.3 | 29.9 | 22.7 |
| 289 | Dhalai | 10.0 | 9.4 | 71.0 | 65.5 |
| 290 | South Garo Hills | 12.4 | 3.9 | 39.8 |  |
| 291 | Ribhoi | 5.0 | 9.6 | 47.2 | 33.7 |
| 292 | East Khasi Hills | 16.6 | 11.2 | 34.3 | 41.0 |
| 293 | Kokrajhar | 14.0 | 10.6 | 61.3 | 65.2 |
| 294 | Goalpara | 12.2 | 11.8 | 69.9 | 82.6 |
| 295 | Barpeta | 12.9 | 11.0 | 65.1 | 60.8 |
| 296 | Morigaon | 13.0 | 9.1 | 69.9 | 73.6 |
| 297 | Lakhimpur | 17.8 | 18.2 | 64.7 | 85.2 |
| 298 | Dhemaji | 17.5 | 13.5 | 69.3 | 80.6 |
| 299 | Tinsukia | 19.9 | 20.9 | 53.1 | 60.8 |
| 300 | Dibrugarh | 20.8 | 17.3 | 59.4 | 68.9 |
| 301 | Golaghat | 7.3 | 16.2 | 60.2 | 74.4 |
| 302 | Dima Hasao | 20.1 | 15.2 | 62.1 | 77.1 |
| 303 | Cachar | 19.2 | 12.2 | 58.4 | 66.7 |
| 304 | Karimganj | 13.8 | 5.2 | 57.2 | 62.2 |
| 305 | Hailakandi | 14.2 | 8.9 | 66.3 | 71.8 |
| 306 | Bongaigaon | 9.0 | 8.6 | 70.1 | 80.2 |
| 307 | Chirang | 10.3 | 8.4 | 61.0 | 73.4 |
| 308 | Kamrup | 27.4 | 26.8 | 63.5 | 72.2 |
| 309 | Kamrup Metropolitan | 28.0 | 24.3 | 67.2 | 70.5 |
| 310 | Nalbari | 20.9 | 25.2 | 65.7 | 82.3 |
| 311 | Baksa | 17.0 | 12.4 | 74.1 | 81.9 |
| 312 | Darrang | 16.3 | 10.1 | 57.8 | 78.6 |
| 313 | Udalguri | 8.8 | 9.5 | 53.5 | 75.8 |
| 314 | Darjiling | 22.1 | 18.1 | 72.8 | 86.4 |
| 315 | Jalpaiguri | 16.3 | 19.1 | 69.4 | 88.3 |
| 316 | Koch Bihar | 14.1 | 15.0 | 81.8 | 89.6 |
| 317 | Uttar Dinajpur | 21.7 | 13.2 | 74.4 | 81.4 |
| 318 | Dakshin Dinajpur | 18.1 | 18.7 | 74.9 | 90.3 |
| 319 | Maldah | 13.6 | 15.2 | 70.6 | 89.0 |
| 320 | Murshidabad | 14.1 | 20.3 | 82.1 | 92.4 |
| 321 | Birbhum | 13.0 | 11.9 | 77.1 | 91.5 |
| 322 | Nadia | 20.6 | 30.2 | 83.0 | 92.3 |
| 323 | North Twenty Four Parganas | 26.2 | 34.9 | 82.7 | 90.7 |
| 324 | Hugli | 28.3 | 33.6 | 74.7 | 87.9 |
| 325 | Bankura | 15.7 | 18.7 | 69.4 | 72.7 |
| 326 | Puruliya | 8.0 | 6.9 | 68.6 | 84.0 |
| 327 | South Twenty Four Parganas | 22.7 | 21.3 | 61.4 | 79.1 |
| 328 | Paschim Medinipur | 15.8 | 15.9 | 73.9 | 75.8 |
| 329 | Purba Medinipur | 25.8 | 26.9 | 62.2 | 81.0 |
| 330 | Garhwa | 5.6 | 2.7 | 33.9 | 49.1 |
| 331 | Chatra | 4.4 | 4.1 | 39.9 | 56.1 |
| 332 | Kodarma | 4.1 | 5.4 | 46.9 | 61.5 |
| 333 | Giridih | 4.6 | 3.6 | 34.5 | 41.2 |
| 334 | Deoghar | 2.7 | 2.8 | 38.8 | 47.0 |
| 335 | Godda | 5.8 | 2.9 | 48.7 | 40.1 |
| 336 | Sahibganj | 4.6 | 3.8 | 36.6 | 48.5 |
| 337 | Pakur | 3.3 | 3.2 | 45.7 | 47.8 |
| 338 | Dhanbad | 13.7 | 6.2 | 38.1 | 42.5 |
| 339 | Bokaro | 6.4 | 9.9 | 42.3 | 43.4 |
| 340 | Lohardaga | 4.9 | 4.9 | 35.4 | 53.3 |
| 341 | Purbi Singhbhum | 6.7 | 9.2 | 43.9 | 39.9 |
| 342 | Palamu | 4.4 | 6.8 | 49.4 | 55.7 |
| 343 | Latehar | 6.0 | 7.2 | 48.7 | 49.4 |
| 344 | Hazaribagh | 7.1 | 8.2 | 43.3 | 38.8 |
| 345 | Ramgarh | 6.1 | 6.0 | 49.2 | 58.9 |
| 346 | Dumka | 3.8 | 3.4 | 47.6 | 65.6 |
| 347 | Jamtara | 4.6 | 6.4 | 39.9 | 48.8 |
| 348 | Ranchi | 13.8 | 8.9 | 42.7 | 48.3 |
| 349 | Khunti | 7.1 | 3.3 | 35.0 | 50.8 |
| 350 | Gumla | 6.4 | 5.1 | 51.0 | 54.3 |
| 351 | Simdega | 6.5 | 2.3 | 42.2 | 53.9 |
| 352 | Pashchimi Singhbhum | 3.8 | 3.2 | 46.4 | 46.6 |
| 353 | Saraikela-Kharsawan | 5.9 | 5.9 | 38.2 | 42.3 |
| 354 | Bargarh | 21.0 | 22.4 | 66.1 | 73.0 |
| 355 | Jharsuguda | 19.8 | 17.0 | 60.0 | 63.1 |
| 356 | Sambalpur | 23.2 | 16.5 | 53.8 | 74.7 |
| 357 | Debagarh | 9.8 | 8.7 | 49.0 | 61.3 |
| 358 | Sundargarh | 9.0 | 13.5 | 63.1 | 72.3 |
| 359 | Kendujhar | 15.8 | 9.3 | 42.4 | 71.4 |
| 360 | Mayurbhanj | 10.8 | 7.9 | 55.8 | 71.0 |
| 361 | Baleshwar | 14.4 | 24.9 | 62.2 | 79.2 |
| 362 | Bhadrak | 13.8 | 12.2 | 68.8 | 83.4 |
| 363 | Kendrapara | 11.4 | 15.3 | 50.4 | 66.7 |
| 364 | Cuttack | 23.7 | 18.5 | 54.8 | 72.5 |
| 365 | Jajapur | 13.2 | 12.7 | 61.6 | 77.2 |
| 366 | Dhenkanal | 18.3 | 17.7 | 61.6 | 69.4 |
| 367 | Anugul | 7.4 | 8.9 | 51.3 | 66.3 |
| 368 | Nayagarh | 12.4 | 10.5 | 55.6 | 81.2 |
| 369 | Khordha | 16.1 | 20.4 | 63.5 | 75.0 |
| 370 | Puri | 23.8 | 24.7 | 62.0 | 82.6 |
| 371 | Ganjam | 13.2 | 11.5 | 52.7 | 66.2 |
| 372 | Gajapati | 9.7 | 15.2 | 52.9 | 56.7 |
| 373 | Kandhamal | 13.1 | 8.6 | 57.6 | 70.3 |
| 374 | Baudh | 8.7 | 9.2 | 60.0 | 72.4 |
| 375 | Subarnapur | 17.1 | 18.7 | 58.1 | 74.5 |
| 376 | Balangir | 13.8 | 13.6 | 54.2 | 82.6 |
| 377 | Nuapada | 8.3 | 11.9 | 51.1 | 74.3 |
| 378 | Kalahandi | 13.2 | 11.0 | 45.0 | 46.5 |
| 379 | Rayagada | 9.0 | 9.5 | 52.1 | 53.2 |
| 380 | Nabarangapur | 5.7 | 5.6 | 58.3 | 70.2 |
| 381 | Koraput | 6.5 | 5.0 | 59.9 | 54.3 |
| 382 | Malkangiri | 6.7 | 5.7 |  | 62.8 |
| 383 | Koriya | 6.0 | 6.9 | 62.5 | 55.4 |
| 384 | Jashpur | 5.1 | 5.6 | 53.6 | 48.6 |
| 385 | Raigarh | 7.1 | 11.0 | 50.8 | 59.0 |
| 386 | Korba | 6.0 | 8.4 | 40.0 | 61.8 |
| 387 | Janjgir-Champa | 3.1 | 9.9 | 49.1 | 65.7 |
| 388 | Kabeerdham | 5.7 | 2.8 | 57.4 | 51.2 |
| 389 | Rajnandgaon | 6.0 | 4.3 | 35.6 | 59.5 |
| 390 | Mahasamund | 4.1 | 4.7 | 44.0 | 40.3 |
| 391 | Dhamtari | 3.7 | 8.4 | 53.8 | 58.6 |
| 392 | Uttar Bastar Kanker | 4.0 | 5.5 | 51.4 | 52.3 |
| 393 | Narayanpur | 5.0 | 4.0 | 47.5 | 43.5 |
| 394 | Bijapur | 4.8 | 4.9 | 49.2 | 49.1 |
| 395 | Sheopur | 7.1 | 6.9 | 55.4 | 49.1 |
| 396 | Morena | 2.7 | 3.2 | 49.6 | 43.9 |
| 397 | Bhind | 3.4 | 4.4 | 38.8 | 66.9 |
| 398 | Gwalior | 8.9 | 9.6 | 48.1 | 51.6 |
| 399 | Datia | 4.3 | 5.1 | 46.2 | 62.9 |
| 400 | Shivpuri | 7.0 | 4.3 | 51.0 | 62.0 |
| 401 | Tikamgarh | 4.5 | 2.0 | 48.8 | 54.7 |
| 402 | Chhatarpur | 7.2 | 4.0 | 34.8 | 60.3 |
| 403 | Panna | 3.4 | 6.9 | 44.3 | 48.2 |
| 404 | Sagar | 6.8 | 7.5 | 45.7 | 42.6 |
| 405 | Damoh | 4.3 | 5.8 | 49.3 | 57.4 |
| 406 | Satna | 4.7 | 4.6 | 33.9 | 56.3 |
| 407 | Rewa | 5.2 | 7.1 | 36.1 | 60.0 |
| 408 | Umaria | 6.8 | 6.9 | 46.2 | 69.8 |
| 409 | Neemuch | 6.1 | 8.3 | 34.5 | 47.4 |
| 410 | Mandsaur | 11.0 | 11.2 | 36.9 | 72.9 |
| 411 | Ratlam | 9.5 | 6.0 | 38.2 | 57.1 |
| 412 | Ujjain | 5.5 | 7.3 | 42.9 | 53.0 |
| 413 | Dewas | 7.3 | 6.2 | 55.7 | 62.4 |
| 414 | Dhar | 6.0 | 4.2 | 41.4 | 65.3 |
| 415 | Indore | 14.2 | 7.0 | 38.7 | 51.3 |
| 416 | Khargone (West Nimar) | 11.1 | 10.8 | 48.0 | 52.5 |
| 417 | Barwani | 8.4 | 7.3 | 43.7 | 64.6 |
| 418 | Rajgarh | 6.1 | 5.7 | 37.2 | 50.9 |
| 419 | Vidisha | 3.7 | 4.6 | 43.7 | 49.2 |
| 420 | Bhopal | 10.6 | 11.0 | 46.1 | 39.8 |
| 421 | Sehore | 6.7 | 8.6 | 44.0 | 52.0 |
| 422 | Raisen | 6.7 | 9.0 | 44.2 | 60.2 |
| 423 | Betul | 5.3 | 6.4 | 53.1 | 65.4 |
| 424 | Harda | 8.3 | 8.4 | 55.4 | 57.2 |
| 425 | Hoshangabad | 11.4 | 16.3 | 49.6 | 59.4 |
| 426 | Katni | 3.5 | 2.1 | 57.5 | 52.8 |
| 427 | Jabalpur | 9.9 | 3.5 | 53.8 | 75.3 |
| 428 | Narsimhapur | 5.0 | 11.2 | 44.1 | 54.4 |
| 429 | Dindori | 2.6 | 2.4 | 42.8 | 55.9 |
| 430 | Mandla | 4.6 | 5.3 | 49.4 | 73.1 |
| 431 | Chhindwara | 7.7 | 8.5 | 45.7 | 65.4 |
| 432 | Seoni | 4.0 | 8.4 | 44.4 | 75.8 |
| 433 | Balaghat | 9.8 | 11.0 | 58.1 | 75.2 |
| 434 | Guna | 4.0 | 2.2 | 42.0 | 61.1 |
| 435 | Ashoknagar | 3.4 | 1.7 | 47.9 | 63.7 |
| 436 | Shahdol | 11.6 | 6.6 | 47.7 | 57.5 |
| 437 | Anuppur | 5.6 | 11.4 | 46.1 | 57.9 |
| 438 | Singrauli | 4.0 | 2.8 | 43.5 | 46.6 |
| 439 | Jhabua | 3.1 | 4.8 | 39.5 | 48.8 |
| 440 | Alirajpur | 2.3 | 2.9 | 38.3 | 54.0 |
| 441 | Khandwa (East Nimar) | 10.9 | 5.9 | 43.9 | 47.7 |
| 442 | Burhanpur | 9.8 | 6.2 | 45.7 | 54.3 |
| 443 | Kachchh | 10.9 | 8.1 | 26.1 | 23.0 |
| 444 | Banas Kantha | 7.7 | 7.8 | 16.0 | 24.4 |
| 445 | Patan | 11.4 | 12.6 | 27.0 | 19.0 |
| 446 | Mahesana | 13.6 | 11.7 | 35.8 | 35.6 |
| 447 | Gandhinagar | 9.5 | 19.9 | 30.7 | 36.9 |
| 448 | Porbandar | 11.4 | 7.7 | 27.0 | 31.9 |
| 449 | Anand | 11.1 | 8.5 | 34.5 | 32.5 |
| 450 | Dohad | 10.3 | 2.0 | 18.3 | 20.5 |
| 451 | Narmada | 7.7 | 4.1 | 25.8 | 25.8 |
| 452 | Bharuch | 6.1 | 6.9 | 26.5 | 33.1 |
| 453 | The Dangs | 8.1 | 4.9 | 16.7 | 30.0 |
| 454 | Navsari | 12.4 | 15.8 | 31.8 | 31.7 |
| 455 | Valsad | 12.3 | 8.0 | 29.9 | 25.0 |
| 456 | Tapi | 10.0 | 7.8 | 34.6 | 31.1 |
| 457 | Daman | 11.9 | 14.6 | 30.5 | 33.5 |
| 458 | Dadra & Nagar Haveli | 18.1 | 16.1 | 45.8 | 40.3 |
| 459 | Nandurbar | 6.8 | 5.4 | 27.8 | 31.7 |
| 460 | Dhule | 16.8 | 11.8 | 32.0 | 37.8 |
| 461 | Jalgaon | 12.5 | 21.0 | 36.9 | 37.9 |
| 462 | Buldana | 11.2 | 12.8 | 35.2 | 28.1 |
| 463 | Akola | 20.2 | 20.6 | 33.2 | 43.4 |
| 464 | Washim | 11.2 | 13.4 | 27.7 | 29.0 |
| 465 | Amravati | 14.5 | 22.5 | 33.8 | 55.9 |
| 466 | Wardha | 23.8 | 17.7 | 42.1 | 38.7 |
| 467 | Nagpur | 26.3 | 24.2 | 48.5 | 38.0 |
| 468 | Bhandara | 18.6 | 28.2 | 47.5 | 44.5 |
| 469 | Gondiya | 11.6 | 20.3 | 47.1 | 57.5 |
| 470 | Gadchiroli | 8.2 | 12.1 | 36.4 | 62.7 |
| 471 | Chandrapur | 7.9 | 14.4 | 39.9 | 37.7 |
| 472 | Yavatmal | 6.9 | 9.6 | 28.2 | 31.8 |
| 473 | Nanded | 18.3 | 8.6 | 35.7 | 29.2 |
| 474 | Hingoli | 11.1 | 11.5 | 24.7 | 29.4 |
| 475 | Parbhani | 20.6 | 10.8 | 27.1 | 18.2 |
| 476 | Jalna | 9.5 | 7.7 | 29.1 | 30.2 |
| 477 | Nashik | 11.1 | 11.0 | 30.5 | 37.6 |
| 478 | Mumbai Suburban | 12.6 | 22.5 | 35.5 | 40.6 |
| 479 | Mumbai | 15.4 | 20.2 | 33.1 | 39.6 |
| 480 | Raigarh | 18.2 | 15.8 | 32.0 | 36.2 |
| 481 | Pune | 14.1 | 23.9 | 39.6 | 42.3 |
| 482 | Ahmadnagar | 17.9 | 18.2 | 30.9 | 30.8 |
| 483 | Bid | 9.6 | 14.1 | 23.5 | 23.4 |
| 484 | Latur | 10.0 | 11.6 | 33.1 | 32.5 |
| 485 | Osmanabad | 9.5 | 11.7 | 32.3 | 32.1 |
| 486 | Solapur | 11.5 | 9.9 | 35.1 | 35.0 |
| 487 | Satara | 12.2 | 14.4 | 37.8 | 51.1 |
| 488 | Ratnagiri | 17.3 | 21.0 | 34.4 | 34.8 |
| 489 | Sindhudurg | 21.8 | 19.7 | 30.1 | 43.2 |
| 490 | Kolhapur | 13.4 | 16.1 | 45.5 | 55.9 |
| 491 | Sangli | 14.5 | 17.7 | 38.9 | 41.9 |
| 492 | Belgaum | 10.7 | 14.6 | 47.8 | 41.3 |
| 493 | Bagalkot | 11.4 | 13.8 | 35.5 | 61.3 |
| 494 | Bijapur | 11.6 | 9.8 | 48.0 | 46.8 |
| 495 | Bidar | 20.3 | 14.2 | 41.6 | 43.0 |
| 496 | Raichur | 7.5 | 12.7 | 47.6 | 49.4 |
| 497 | Koppal | 9.2 | 12.2 | 45.4 | 47.3 |
| 498 | Gadag | 19.4 | 26.2 | 45.2 | 53.0 |
| 499 | Dharwad | 14.5 | 18.9 | 43.1 | 55.9 |
| 500 | Uttara Kannada | 14.6 | 27.2 | 38.1 | 47.6 |
| 501 | Haveri | 15.3 | 19.1 | 52.0 | 52.0 |
| 502 | Bellary | 18.6 | 18.2 | 53.2 | 71.4 |
| 503 | Chitradurga | 22.5 | 29.1 | 53.7 | 68.9 |
| 504 | Davanagere | 22.3 | 30.9 | 47.6 | 59.4 |
| 505 | Shimoga | 31.0 | 34.9 | 37.4 | 68.0 |
| 506 | Chikmagalur | 31.2 | 30.2 | 47.9 | 62.6 |
| 507 | Tumkur | 25.3 | 42.5 | 56.6 | 61.9 |
| 508 | Bangalore | 17.8 | 16.3 | 41.9 | 51.8 |
| 509 | Mandya | 24.1 | 23.6 | 46.1 | 58.9 |
| 510 | Hassan | 18.7 | 26.6 | 58.2 | 67.9 |
| 511 | Dakshina Kannada | 22.9 | 19.8 | 38.1 | 36.3 |
| 512 | Kodagu | 21.6 | 26.2 | 43.5 | 47.5 |
| 513 | Mysore | 20.3 | 19.2 | 56.3 | 66.5 |
| 514 | Chamarajanagar | 14.4 | 25.0 | 51.1 | 57.2 |
| 515 | Gulbarga | 7.3 | 15.4 | 25.2 | 50.2 |
| 516 | Yadgir | 5.8 | 6.7 | 36.5 | 45.5 |
| 517 | Kolar | 25.6 | 31.5 | 50.7 | 61.6 |
| 518 | Chikkaballapura | 21.3 | 33.2 | 50.0 | 51.3 |
| 519 | Bangalore Rural | 18.8 | 33.8 | 45.6 | 60.0 |
| 520 | Ramanagara | 23.2 | 28.0 | 44.3 | 59.0 |
| 521 | North Goa | 21.3 | 32.9 | 54.3 | 45.6 |
| 522 | South Goa | 14.5 | 21.7 | 58.6 | 53.2 |
| 523 | Wayanad | 23.4 | 21.4 | 29.5 | 33.3 |
| 524 | Kozhikode | 20.5 | 41.9 | 48.2 | 47.5 |
| 525 | Malappuram | 24.2 | 22.7 | 34.6 | 27.1 |
| 526 | Palakkad | 21.1 | 32.9 | 36.8 | 29.5 |
| 527 | Thrissur | 22.6 | 51.7 | 33.2 | 41.7 |
| 528 | Ernakulam | 32.3 | 48.4 | 39.9 | 50.4 |
| 529 | Idukki | 33.1 | 34.6 | 41.0 | 47.2 |
| 530 | Kottayam | 17.6 | 46.0 | 32.0 | 48.0 |
| 531 | Pathanamthitta | 33.8 | 48.3 | 46.0 | 59.7 |
| 532 | Kollam | 27.4 | 40.0 | 51.6 | 56.3 |
| 533 | Thiruvananthapuram | 17.1 | 27.2 | 44.4 | 39.1 |
| 534 | Chennai | 29.6 | 38.2 | 39.3 | 62.0 |
| 535 | Kancheepuram | 27.2 | 31.5 | 47.0 | 62.4 |
| 536 | Vellore | 24.1 | 40.0 | 43.0 | 47.4 |
| 537 | Tiruvannamalai | 17.0 | 22.8 | 45.8 | 70.1 |
| 538 | Viluppuram | 16.2 | 22.8 | 41.4 | 57.4 |
| 539 | Salem | 16.6 | 33.9 | 50.3 | 58.5 |
| 540 | Namakkal | 22.4 | 44.0 | 59.2 | 69.7 |
| 541 | Erode | 21.4 | 32.8 | 50.9 | 72.5 |
| 542 | The Nilgiris | 21.0 | 38.2 | 41.5 | 60.0 |
| 543 | Dindigul | 22.0 | 27.4 | 50.9 | 60.4 |
| 544 | Karur | 29.1 | 35.3 | 52.3 | 67.1 |
| 545 | Tiruchirappalli | 22.2 | 29.9 | 51.7 | 67.0 |
| 546 | Perambalur | 39.2 | 40.4 | 55.9 | 73.6 |
| 547 | Ariyalur | 29.7 | 48.7 | 56.5 | 79.7 |
| 548 | Cuddalore | 33.1 | 45.0 | 54.1 | 68.5 |
| 549 | Nagapattinam | 39.0 | 41.1 | 51.4 | 73.3 |
| 550 | Thiruvarur | 29.5 | 47.1 | 68.1 | 78.8 |
| 551 | Thanjavur | 36.2 | 33.8 | 59.1 | 76.1 |
| 552 | Pudukkottai | 30.4 | 30.3 | 56.5 | 70.7 |
| 553 | Sivaganga | 35.1 | 44.4 | 61.7 | 49.8 |
| 554 | Madurai | 32.8 | 32.6 | 51.1 | 67.5 |
| 555 | Theni | 19.6 | 40.0 | 62.2 | 62.6 |
| 556 | Virudhunagar | 42.4 | 43.9 | 53.9 | 68.5 |
| 557 | Thoothukkudi | 37.0 | 38.2 | 54.4 | 72.1 |
| 558 | Tirunelveli | 32.5 | 54.8 | 55.1 | 68.2 |
| 559 | Kanniyakumari | 37.7 | 52.2 | 55.3 | 77.4 |
| 560 | Dharmapuri | 24.0 | 19.3 | 59.2 | 60.7 |
| 561 | Krishnagiri | 18.6 | 22.7 | 52.4 | 63.8 |
| 562 | Coimbatore | 27.6 | 37.0 | 56.9 | 72.8 |
| 563 | Tiruppur | 32.9 | 37.7 | 45.2 | 56.8 |
| 564 | Yanam | 21.4 | 43.3 | 61.9 | 76.8 |
| 565 | Puducherry | 30.8 | 30.3 | 45.3 | 46.9 |
| 566 | East Siang | 6.7 | 14.6 | 35.9 | 63.9 |
| 567 | Kra Daadi | 8.0 | 25.2 | 30.4 | 45.0 |
| 568 | Kurung Kumey | 7.9 | 22.0 | 30.3 | 33.3 |
| 569 | Lohit | 8.3 | 15.1 | 41.0 | 63.9 |
| 570 | Longding | 5.0 | 4.9 |  | 56.1 |
| 571 | Namsai | 8.1 | 11.1 | 41.1 | 64.2 |
| 572 | Siang | 8.0 | 6.5 | 33.7 | 50.3 |
| 573 | Tirap | 5.1 | 22.2 | 37.4 | 64.6 |
| 574 | West Siang | 12.0 | 18.1 | 33.7 | 59.5 |
| 575 | Biswanath | 13.8 | 9.7 | 55.3 | 79.3 |
| 576 | Charaideo | 16.6 | 14.3 | 59.0 | 68.0 |
| 577 | Dhubri | 8.3 | 7.1 | 61.3 | 77.5 |
| 578 | Hojai | 10.5 | 9.6 | 60.8 | 58.5 |
| 579 | Jorhat | 24.0 | 31.0 | 64.3 | 85.0 |
| 580 | Karbi Anglong | 10.6 | 14.0 | 59.3 | 71.8 |
| 581 | Majuli | 23.1 | 19.1 |  | 79.6 |
| 582 | Nagaon | 11.1 | 11.5 | 60.8 | 80.5 |
| 583 | Sivasagar | 17.6 | 18.7 | 59.1 | 77.9 |
| 584 | Sonitpur | 13.8 | 15.4 | 55.3 | 73.5 |
| 585 | South Salmara Mancachar | 8.2 | 5.0 |  | 71.1 |
| 586 | West Karbi Anglong | 11.6 | 5.5 | 59.4 | 67.6 |
| 587 | Balod | 8.7 | 9.6 | 43.2 | 56.3 |
| 588 | Baloda Bazar | 9.5 | 2.5 | 57.4 | 63.2 |
| 589 | Balrampur | 4.8 | 2.1 | 38.6 | 33.0 |
| 590 | Bastar | 3.2 | 4.8 | 45.3 | 49.3 |
| 591 | Bemetara | 8.7 | 5.3 | 43.3 | 63.8 |
| 592 | Bilaspur | 3.8 | 7.1 | 47.7 | 60.2 |
| 593 | Dantewada | 6.6 | 3.8 | 51.4 | 43.0 |
| 594 | Durg | 9.1 | 12.5 | 43.3 | 61.4 |
| 595 | Gariyaband | 8.8 | 6.9 | 57.4 | 58.9 |
| 596 | Kodagaon | 3.1 | 2.4 | 45.3 | 41.1 |
| 597 | Mungeli | 3.8 | 2.0 | 47.6 | 66.8 |
| 598 | Raipur | 9.8 | 10.7 | 57.4 | 50.5 |
| 599 | Sukma | 6.5 | 3.3 | 51.4 | 57.2 |
| 600 | Surguja | 8.8 | 4.4 | 51.2 | 51.7 |
| 601 | Central | 15.6 | 18.8 | 40.8 | 48.0 |
| 602 | East | 25.1 | 16.8 | 45.4 | 47.7 |
| 603 | New Delhi | 13.3 | 17.9 | 45.9 | 44.9 |
| 604 | North | 18.0 | 12.7 | 33.8 | 43.3 |
| 605 | North East | 19.9 | 19.2 | 44.8 | 44.7 |
| 606 | North West | 16.0 | 18.2 | 34.4 | 34.1 |
| 607 | Shahdara | 22.4 | 16.8 | 45.2 | 40.6 |
| 608 | South | 26.2 | 15.4 | 48.7 | 47.8 |
| 609 | South East | 26.3 | 17.6 | 48.1 | 34.3 |
| 610 | South West | 19.0 | 14.3 | 48.2 | 51.1 |
| 611 | West | 22.9 | 14.2 | 45.3 | 45.5 |
| 612 | Ahmadabad | 23.3 | 20.7 | 46.4 | 41.2 |
| 613 | Aravali | 10.6 | 11.4 | 21.9 | 28.9 |
| 614 | Bhavnagar | 10.3 | 13.6 | 20.4 | 25.6 |
| 615 | Botad | 9.4 | 6.2 | 20.4 | 23.4 |
| 616 | Chhota Udaipur | 8.0 | 4.9 | 38.3 | 20.3 |
| 617 | Devbhumi Dwarka | 10.8 | 8.1 | 33.7 | 23.1 |
| 618 | Gir Somnath | 11.8 | 8.4 | 31.8 | 22.2 |
| 619 | Jamnagar | 11.0 | 13.8 | 33.7 | 30.5 |
| 620 | Junagadh | 13.2 | 13.1 | 31.8 | 30.1 |
| 621 | Kheda | 9.7 | 6.5 | 23.1 | 30.0 |
| 622 | Mahisagar | 8.6 | 9.9 | 21.7 | 18.8 |
| 623 | Morbi | 8.0 | 5.0 | 22.4 | 27.7 |
| 624 | Panch Mahals | 7.4 | 4.0 | 20.6 | 14.9 |
| 625 | Rajkot | 8.7 | 9.8 | 23.1 | 31.6 |
| 626 | Sabar Kantha | 10.8 | 9.7 | 21.9 | 35.5 |
| 627 | Surendranagar | 7.8 | 5.5 | 21.4 | 23.6 |
| 628 | Vadodara | 8.5 | 12.8 | 38.3 | 41.6 |
| 629 | Bhiwani | 8.0 | 6.9 | 20.4 | 19.2 |
| 630 | Charkhi Dadri | 8.2 | 9.4 | 20.4 | 20.1 |
| 631 | Agar Malwa | 3.8 | 7.1 | 46.6 | 75.7 |
| 632 | Shajapur | 3.9 | 5.7 | 46.6 | 52.6 |
| 633 | Palghar | 11.3 | 9.7 | 38.8 | 48.9 |
| 634 | Thane | 11.9 | 15.7 | 38.8 | 40.0 |
| 635 | East Garo Hills | 11.5 | 4.3 |  | 48.1 |
| 636 | East Jantia Hills | 4.9 | 6.9 | 38.1 | 37.1 |
| 637 | North Garo Hills | 11.2 | 4.9 | 47.4 | 26.1 |
| 638 | South West Garo Hills | 7.6 | 3.1 | 42.1 |  |
| 639 | South West Khasi Hills | 8.1 | 6.1 | 34.7 | 33.2 |
| 640 | West Garo Hills | 7.7 | 7.1 | 42.1 | 27.2 |
| 641 | West Jaintia Hills | 5.0 | 7.6 | 38.2 | 35.6 |
| 642 | West Khasi Hills | 7.3 | 7.2 | 34.8 | 30.1 |
| 643 | Fazilka | 11.8 | 17.0 | 30.9 | 41.7 |
| 644 | Firozpur | 12.0 | 26.2 | 31.0 | 55.9 |
| 645 | Gurdaspur | 22.4 | 32.9 | 48.9 | 67.9 |
| 646 | Pathankot | 20.4 | 36.7 | 48.9 | 72.5 |
| 647 | Adilabad | 40.3 | 33.1 | 76.5 | 78.7 |
| 648 | Bhadradri Kothagudem | 49.1 | 49.9 | 78.6 | 77.5 |
| 649 | Hyderabad | 30.8 | 39.5 | 66.6 | 85.9 |
| 650 | Jagitial |  | 66.9 | 82.8 | 81.6 |
| 651 | Jangoan | 58.5 | 69.8 | 81.7 | 86.7 |
| 652 | Jayashankar Bhupalapally | 53.8 | 50.9 | 82.0 | 87.3 |
| 653 | Jogulamba Gadwal | 26.0 | 21.3 | 70.8 | 71.7 |
| 654 | Kamareddy | 33.1 | 41.0 | 76.4 | 81.5 |
| 655 | Karimnagar | 59.7 | 66.7 | 82.8 | 91.9 |
| 656 | Khammam | 50.8 | 52.4 | 78.7 | 80.6 |
| 657 | Komaram Bheem Asifabad |  | 18.6 | 76.5 | 75.7 |
| 658 | Mahabubabad | 55.2 | 59.2 | 80.9 | 86.6 |
| 659 | Mahabubnagar | 26.4 | 25.4 | 70.8 | 76.2 |
| 660 | Mancherial | 45.1 | 45.9 | 76.6 | 91.4 |
| 661 | Medak | 36.2 | 40.2 | 71.2 | 81.0 |
| 662 | Medchal-Malkajgiri | 35.5 | 45.8 | 76.4 | 78.9 |
| 663 | Nagarkurnool | 29.6 | 42.4 | 70.8 | 79.0 |
| 664 | Nalgonda | 47.8 | 52.8 | 80.8 | 89.7 |
| 665 | Nirmal | 44.6 | 58.9 | 76.6 | 91.3 |
| 666 | Nizamabad | 35.5 | 48.3 | 76.4 | 89.9 |
| 667 | Peddapalli | 61.8 | 66.4 | 82.8 | 89.2 |
| 668 | Rajanna Sircilla | 44.9 | 61.6 | 80.9 | 90.6 |
| 669 | Ranga Reddy | 28.7 | 39.4 | 74.5 | 77.8 |
| 670 | Sangareddy | 31.2 | 35.6 | 71.2 | 76.3 |
| 671 | Siddipet | 35.6 | 58.3 | 74.1 | 82.9 |
| 672 | Suryapet | 46.7 | 65.8 | 80.8 | 89.5 |
| 673 | Vikarabad | 36.1 | 35.0 | 76.4 | 73.7 |
| 674 | Wanaparthy | 24.5 | 35.1 | 70.8 | 73.3 |
| 675 | Warangal Rural | 55.4 | 64.9 | 81.8 | 86.4 |
| 676 | Warangal Urban | 54.2 | 44.9 | 81.7 | 89.3 |
| 677 | Yadadri Bhuvanagiri | 47.3 | 61.6 | 80.8 | 83.5 |
| 678 | Gomati | 8.1 | 18.9 | 72.5 | 70.9 |
| 679 | Khowai | 29.8 | 24.0 | 79.8 | 79.7 |
| 680 | North Tripura | 12.5 | 13.1 | 52.7 | 47.6 |
| 681 | South Tripura | 7.9 | 14.1 | 72.5 | 78.6 |
| 682 | Unakoti | 11.9 | 8.7 | 52.7 | 49.5 |
| 683 | West Tripura | 33.2 | 35.7 | 79.8 | 84.0 |
| 684 | Amethi | 3.7 | 3.6 | 32.6 | 38.6 |
| 685 | Budaun | 4.0 | 3.4 | 30.8 | 24.4 |
| 686 | Ghaziabad | 13.4 | 11.3 | 36.8 | 46.8 |
| 687 | Hapur | 13.5 | 5.9 | 36.6 | 30.6 |
| 688 | Moradabad | 5.0 | 8.6 | 41.8 | 41.2 |
| 689 | Muzaffarnagar | 10.1 | 14.8 | 27.9 | 34.3 |
| 690 | Rae Bareli | 3.3 | 3.3 | 40.9 | 48.8 |
| 691 | Sambhal | 4.8 | 1.7 | 40.8 | 25.5 |
| 692 | Shamli | 10.1 | 3.8 | 27.9 | 34.2 |
| 693 | Sultanpur | 3.7 | 4.6 | 29.4 | 42.8 |
| 694 | Paschim Barddhaman | 15.3 | 23.3 | 76.8 | 74.0 |
| 695 | Purba Barddhaman | 14.8 | 19.7 | 76.8 | 88.7 |
| 696 | Thiruvallur | 31.6 | 37.9 | 51.1 | 67.0 |
| 697 | Surat | 17.2 | 10.7 | 30.8 | 32.8 |
| 698 | Amreli | 7.5 | 8.7 | 20.7 | 32.4 |
| 699 | Surajpur | 8.7 | 2.6 | 51.2 | 52.6 |
| 700 | Sidhi | 4.1 | 5.1 | 43.2 | 61.1 |
| 701 | Kheri | 5.1 | 5.2 | 46.6 | 61.6 |
| 702 | Sepahijala | 29.3 | 19.5 | 79.8 | 71.0 |
| 703 | Udupi | 23.6 | 34.2 | 57.2 | 59.8 |
| 704 | Kasaragod | 17.2 | 27.2 | 35.5 | 29.8 |
| 705 | Lakshadweep | 32.9 | 25.1 | 50.7 | 41.3 |
| 706 | Kannur | 20.2 | 34.9 | 32.8 | 41.6 |
| 707 | Alappuzha | 24.0 | 39.9 | 42.4 | 53.1 |
| 708 | Saiha | 6.7 | 8.4 | 49.5 | 43.5 |
| 709 | South Andaman | 22.8 | 25.7 | 63.5 | 80.0 |
| 710 | Ramanathapuram | 38.3 | 41.7 | 51.3 | 62.6 |
| 711 | Nicobars | 6.6 | 9.4 |  | 80.6 |
| 712 | Karaikal | 39.1 | 45.2 | 68.9 | 58.1 |
| 713 | Haora | 17.6 | 26.7 | 77.0 | 72.9 |
| 714 | Jagatsinghapur | 15.8 | 28.1 | 57.8 | 81.6 |
| 715 | North & Middle Andaman | 14.9 | 11.2 | 62.1 | 71.9 |
| 716 | Kargil | 13.1 | 32.2 | 71.8 |  |
| 717 | Aurangabad | 6.0 | 2.2 | 48.0 | 50.5 |
| 718 | Aurangabad | 9.7 | 16.5 | 30.0 | 21.3 |
| 719 | Diu | 11.8 | 24.1 | 49.2 | 48.6 |
| 720 | Mahe | 42.9 | 16.2 | 54.8 | 41.1 |

**Fig B. Maps for district-level percentage change for cesarean section births public and private facilities, India, NFHS 2016 & 2021**

| 1. **Public**   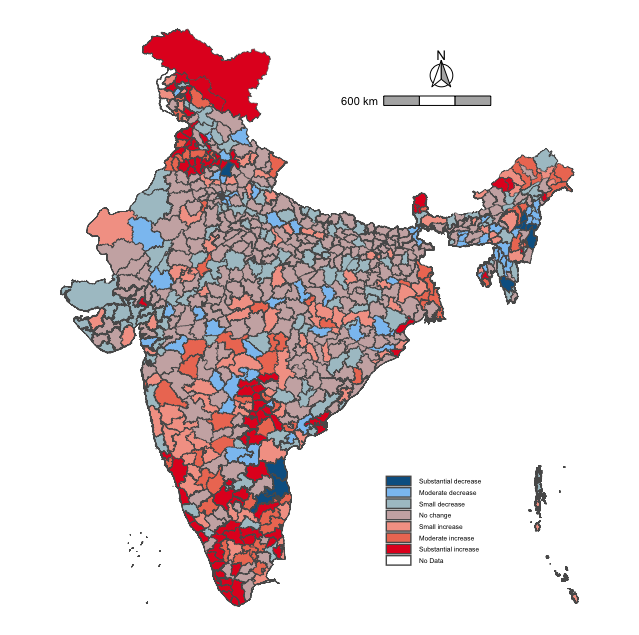 | 1. **Private**   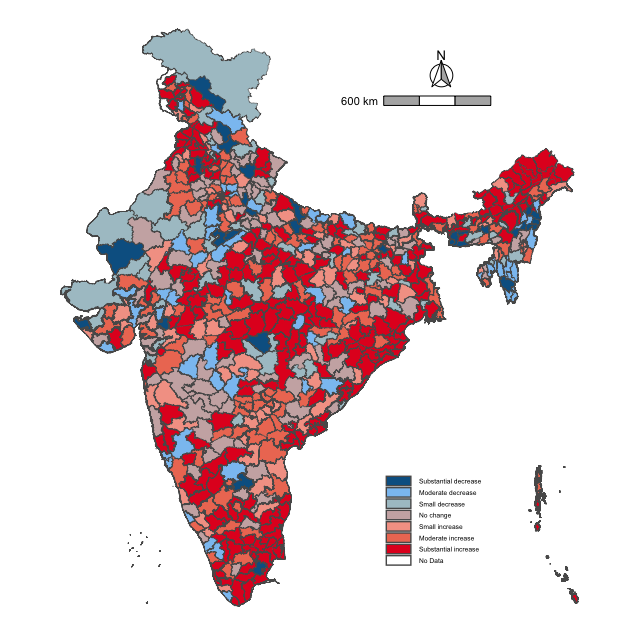 |
| --- | --- |

Note: Cut-points for change (2021 – 2016): Substantial Decrease (>10.00%, dark blue), Moderate Decrease (5.00-9.99%, blue), Small Decrease (2.50-4.99%, light blue), No Change (-1.99 to 1.99%, reddish gray), Small Increase (2.00-4.99%, light red), Moderate Increase (5.00-9.99%, red), Substantial Increase (>10.00%, dark red)
